# Novel enhancers of guanylyl cyclase‐A activity acting via allosteric modulation

**DOI:** 10.1111/bph.16203

**Published:** 2023-08-29

**Authors:** Henriette Andresen, Cristina Pérez‐Ternero, Jerid Robinson, Deborah M. Dickey, Adrian J. Hobbs, Lincoln R. Potter, Finn Olav Levy, Alessandro Cataliotti, Lise Román Moltzau

**Affiliations:** ^1^ Department of Pharmacology, Institute of Clinical Medicine University of Oslo and Oslo University Hospital Oslo Norway; ^2^ William Harvey Research Institute, Barts & The London School of Medicine and Dentistry Queen Mary University of London London UK; ^3^ Department of Biochemistry, Molecular Biology, and Biophysics University of Minnesota Medical School Minneapolis Minnesota USA; ^4^ Institute for Experimental Medical Research University of Oslo and Oslo University Hospital Oslo Norway

**Keywords:** allosteric modulation, cardiovascular pharmacology, drug discovery/target validation, guanylyl cyclase, natriuretic peptides

## Abstract

**Background and Purpose:**

Guanylyl cyclase‐A (GC‐A), activated by endogenous atrial natriuretic peptide (ANP) and brain natriuretic peptide (BNP), plays an important role in the regulation of cardiovascular and renal homeostasis and is an attractive drug target. Even though small molecule modulators allow oral administration and longer half‐life, drug targeting of GC‐A has so far been limited to peptides. Thus, in this study we aimed to develop small molecular activators of GC‐A.

**Experimental Approach:**

Hits were identified through high‐throughput screening and optimized by in silico design. Cyclic GMP was measured in QBIHEK293A cells expressing GC‐A, GC‐B or chimerae of the two receptors using AlphaScreen technology. Binding assays were performed in membrane preparations or whole cells using ^125^I‐ANP. Vasorelaxation was measured in aortic rings isolated from Wistar rats.

**Key Results:**

We have identified small molecular allosteric enhancers of GC‐A, which enhanced ANP or BNP effects in cellular systems and ANP‐induced vasorelaxation in rat aortic rings. The mechanism of action appears novel and not mediated through previously described allosteric binding sites. In addition, the selectivity and activity depend on a single amino acid residue that differs between the two similar receptors GC‐A and GC‐B.

**Conclusion and Implications:**

We describe a novel allosteric binding site on GC‐A, which can be targeted by small molecules to enhance ANP and BNP effects. These compounds will be valuable tools in further development and proof‐of‐concept of GC‐A enhancement for the potential use in cardiovascular therapy.

## Graphical abstract

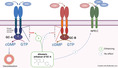

**Abbreviations**
ANP: atrial natriuretic peptide
BNP: brain natriuretic peptide
CCD: coiled‐coil domain/dimerization domain
CNP: C‐type natriuretic peptide
GC‐A: guanylyl cyclase A/natriuretic peptide receptor A
GC‐B: guanylyl cyclase B/natriuretic peptide receptor B
HSP90: heat shock protein 90
KHD: kinase homology domain
NP: natriuretic peptide
NPR‐C: natriuretic peptide receptor C/clearance receptor
U46619: 9,11‐dideoxy‐11α,9α‐epoxymethano‐prostaglandin F_2α_

**What is already known**

- The natriuretic peptide system has become an attractive therapeutic target for cardiovascular diseases.
- Peptides have been developed but with disadvantages of short half‐life and inconvenient administration.

**What does this study add**

- We discovered novel allosteric enhancers that increase the effects of natriuretic peptides on GC‐A.
- We identified a new allosteric binding site on GC‐A useful for further drug development.

**What is the clinical significance**

- The natriuretic peptide system is a validated therapeutic target for different cardiovascular diseases.
- Small molecular compounds, compared to peptides, have greater potential of oral administration and longer half‐life.

## 1 INTRODUCTION

Natriuretic peptides (NPs) and their receptors are important regulators of cardiovascular and renal homeostasis, and enhancement of the activity of the NP system has become an attractive therapeutic target. Endogenous and several synthetic natriuretic peptides have been tested in the treatment of conditions such as heart failure (Kawakami et al., 2018; O'Connor et al., 2011; Packer et al., 2017) and hypertension (Cataliotti et al., 2012; Chen et al., 2020), but the development of small molecular compounds has been challenging, and only a few have been developed to target this system (Iwaki, Nakamura, et al., 2017; Iwaki, Oyama, et al., 2017; Iwaki, Tanaka, et al., 2017; Sangaralingham et al., 2021).

The NP system comprises three distinct hormones, two guanylyl cyclase‐linked receptors and one clearance receptor with G‐protein signalling. Atrial natriuretic peptide (ANP) and brain natriuretic peptide (BNP) activate guanylyl cyclase (GC)‐A (also known as NPR‐A or NPR1) and C‐type natriuretic peptide (CNP) activates GC‐B (also known as NPR‐B or NPR2). The activation of these two receptors produces the second messenger, 3′,5′‐cyclic guanosine monophosphate (cGMP). In the heart and kidneys, GC‐A activation has anti‐remodelling effects as it inhibits hypertrophy and fibrosis. In the kidneys, GC‐A activation also causes natriuresis and diuresis and enhances glomerular filtration rate. GC‐A activation also inhibits the renin‐angiotensin‐aldosterone system (RAAS) in several tissues (Potter et al., 2006). GC‐A activation in the vasculature causes vasodilation, which, together with the renal actions and RAAS inhibition, reduces blood volume and pressure (Holtwick et al., 2002; Lopez et al., 1997). In adipose tissue, GC‐A promotes glucose uptake (Coué et al., 2018) and lipolysis (Sengenès et al., 2000). In animal models, genetic depletion of GC‐A (Lopez et al., 1995; Oliver et al., 1997), ANP (John et al., 1995; John et al., 1996) or BNP (Holditch et al., 2015) leads to hypertension, cardiac hypertrophy/fibrosis and organ damage, demonstrating the importance of GC‐A activation in cardiovascular and renal homeostasis. In humans, genetic studies have demonstrated a link between altered GC‐A function and blood pressure (Vandenwijngaert et al., 2019) and the increased risk of development of hypertension (Nakayama et al., 2000). Early corollary studies demonstrated that people with low NP concentrations had higher blood pressure than those with normal levels of NP (Belluardo et al., 2006; Newton‐Cheh et al., 2009), which suggested the rationale for the development of therapeutic strategies aimed at enhancing this protective hormonal system.

GC‐A is a homodimer with one NP binding site in the extracellular interface between the monomers (He et al., 2001), which results in a ligand to receptor stoichiometry of 1:2. It is hypothesized that binding of NPs induces a small rotation of the two monomers that propagates through the single transmembrane spanning domain, the kinase homology domain (KHD) and the dimerization domain (CCD) to the GC catalytic domain. This leads to increased cGMP production as the two GC domains are brought closer together (Misono et al., 2011; Ogawa et al., 2004). Besides the NP binding domain, several allosteric binding sites have been described on GC‐A (De Léan, 1986; Jewett et al., 1993; Misono, 2000; Ogawa et al., 2010; Poirier et al., 2002; Robinson & Potter, 2011; Robinson & Potter, 2012). In addition to allosteric modulation, the activity of GC‐A is affected by its phosphorylation state (Edmund et al., 2019; Potter & Hunter, 1998) and post‐translational glycosylation (Heim et al., 1996).

Recombinant ANP and BNP have been used to target GC‐A in the treatment of acute heart failure (Tanaka et al., 2018). However, both these peptides also bind to NPR‐C (also known as NPR3), which, in addition to having an intrinsic signalling capacity on its own (Anand‐Srivastava et al., 1996; Rose & Giles, 2008; Tanaka et al., 2018), is known as a “clearance receptor” that internalizes and leads to lysosomal degradation of all NPs. Internalization through NPR‐C and rapid enzymatic cleavage by neprilysin and other proteases are responsible for the short half‐life of these peptides (Dickey & Potter, 2011a). Therefore, researchers have pursued ways to increase the half‐life by designing NPs that have increased resistance to neprilysin degradation (Chen et al., 2018; Dickey & Potter, 2011a) or by inhibiting neprilysin. Valsartan/sacubitril is a combination of the angiotensin receptor blocker valsartan and the neprilysin inhibitor sacubitril and has been shown to be effective in the treatment of chronic heart failure (McMurray et al., 2014). Although the NP system is a validated therapeutic target, valsartan/sacubitril is currently the only small molecular compound on the market that modulates the NP system. This indicates that activation of GC‐A with small molecules is challenging, and to our knowledge, only Iwaki et al. have successfully developed GC‐A agonists that mimic ANP and BNP (Iwaki, Nakamura, et al., 2017; Iwaki, Oyama, et al., 2017; Iwaki, Tanaka, et al., 2017). Small molecular compounds like these have, in contrast to peptides, the potential of oral administration and longer half‐life.

Here, we describe novel allosteric enhancers of GC‐A that are not competitive with ANP or BNP but require activation of GC‐A for their effects. Furthermore, we have explored their mechanism of action and have discovered that they do not modulate the affinity of NPs, but increase their efficacy on GC‐A. Additionally, by exploiting the selectivity of one of these compounds, we have identified a key amino acid in GC‐A that is necessary for the enhancing actions, and we suggest a new allosteric binding site on GC‐A that may be useful for further drug development. After initial submission of our manuscript, a different series of allosteric modulators of GC‐A was also reported, however with notable differences in structure and mechanism of action (Sangaralingham et al., 2021).

## 2 METHODS

### 2.1 Cell cultures

QBIHEK293A (RRID:CVCL_6910), HEK293T (RRID:CVCL_0063) and HeLa (RRID:CVCL_0030) cells were grown in Dulbecco's modified Eagle's medium (DMEM) (Gibco®, ThermoFischer Scientific) that was supplemented with 10% fetal bovine serum (FBS), 100 U·ml^−1^ penicillin and 0.1 mg·ml^−1^ streptomycin. QBIHEK293A cells were stably transfected with codon optimized human GC‐A or GC‐B, as previously described (Bach et al., 2014). For the stable GC‐A‐ and GC‐B‐expressing cells, 1 mg·ml^−1^ geneticin (G418) was added as the selection antibiotic.

### 2.2 The AlphaScreen assay for cGMP

The AlphaScreen assay for cGMP (PerkinElmer, cat. # 6760308) was performed as previously described (Bach et al., 2014). Briefly, QBIHEK293A cells that expressed GC‐A and GC‐B were split the day before the experiment and harvested using a solution of EDTA (Versene, Invitrogen, ThermoFischer Scientific). Cells (6000 per well GC‐A‐expressing, 8000 per well GC‐B‐expressing) were resuspended in stimulation buffer (5 mmol.L^−1^ HEPES in Hanks' balanced salt solution [HBSS] at pH 7.4, 0.1% bovine serum albumin [BSA] with isobutyl‐methyl xanthine [IBMX; 0.7 mmol.L_−1_ final]). Compounds were dissolved in stimulation buffer and added to wells in the indicated concentrations. Cells were incubated with the indicated concentrations of compounds for 20 min in order to make sure the compounds were in binding equilibrium with their target before adding agonists (human BNP, CNP or proBNP) in various concentrations. Cells were stimulated for 20 min with agonist before the reactions were stopped and cells lysed by adding the AlphaScreen Acceptor bead mix (15.6 μg·ml^−1^ AlphaScreen Protein A‐coated Acceptor Beads [final concentration 3.13 μg·ml^−1^], anti‐cGMP antibody [PerkinElmer rabbit polyclonal anti‐cGMP antibody: final dilution 1:8000, Genscript anti‐cGMP antibody, Cat# A00615, RRID:AB_1237577: final dilution 1:50,000] and 0.5% Tween‐20 in 5 mmol·L^−1^ HEPES buffer at pH 7.4). After incubation for 1 h, the Donor bead mix (7.8 μg·ml^−1^ AlphaScreen Streptavidin Donor beads [final concentration 3.13 μg·ml^−1^], biotinylated cGMP [PerkinElmer biotinylated cGMP: 0.625 nM final concentration, BIOLOG biotinylated cGMP: 6.25 nmol·L^−1^ final concentration] and 0.5% Tween‐20 in 5 mmol·L^−1^ HEPES buffer at pH 7.4) was added (40 μl final volume), and incubation continued for 2 h. The luminescence signals were quantified on an EnVision® multilabel plate reader (PerkinElmer) using AlphaScreen emission 570 nm filter. When transiently transfected cells were used, QBIHEK293A cells were grown to 70%–80% confluency and transfected using Lipofectamine LTX Plus (Invitrogen) according to the manufacturer's instructions or by use of polyethyleneimine (PEI) in a 3:1 PEI:DNA ratio. After 48 h, cells (16,000 per well) were harvested using TrypLE™ Select Enzyme (Gibco®, ThermoFisher Scientific), and the assay was carried out as described above. All test compounds were dissolved in DMSO, the concentration of which was kept constant or included in controls for all experiments. Data were analysed using GraphPad Prism 8.3.0 software. For the construction of concentration–response curves for NPs, curves were fitted using non‐linear regression and the built‐in log (agonist) versus response (three parameters) (Hill slope = 1). To construct concentration–response curves for the compounds in the presence of a small concentration of NP, the curves were fitted using log (agonist) versus response – variable slope (four parameters) in GraphPad Prism 8.3.0. To compensate for variable cGMP production between assays, the level of cGMP was normalized to the maximum NP‐mediated cGMP level in each experiment and expressed as percent of control (concentration–response NP) or percent of maximum NP stimulation (concentration–response compound).

### 2.3 High throughput screening

Chemical libraries from Enamine (28,500 compounds) and a protein–protein interaction library from Asinex (BioDesign Library; 1008 compounds) were screened using the AlphaScreen assay for cGMP. Compounds (10 μmol·L^−1^ final concentration in 40 μl) were printed on the plates by a Labcyte Echo 550 Acoustic Liquid Handler. Cells (8000 per well) were added by a Hamilton Microlab Star automated liquid‐handling robot and incubated for 20 min before they were stimulated with a small concentration of rat BNP (EC_10_; 3 nmol·L^−1^). This concentration was chosen to enable the identification of both agonists and allosteric modulators. Maximum cGMP production was induced by the addition of 300 nmol·L^−1^ rat BNP as a control to every plate. Reagents were added with PerkinElmer FlexDrop PLUS to a total volume of 40 μl. Plates were read on a PerkinElmer EnVision® multilabel plate reader using Turbo option.

### 2.4 In silico design

Based on the hit from the high throughput screening, a similarity and substructure search based on the structure of compound #2 was carried out using the databases from MolPort and eMolecules. This was done in two steps, and compounds #1–104 (Table S1) were obtained by in silico design and tested at 10 μM in presence of 3 nM, 40 nM and 300 nM rat BNP in AlphaScreen assays as described above. Active compounds were further tested in a concentration‐dependent manner.

### 2.5 Membrane preparation and binding assay

#### 2.5.1 Membrane preparation

GC‐A‐expressing QBIHEK293A cells were harvested in ice‐cold HBSS and collected by centrifugation (800 × *g*, 5 min, 4°C). The pellet was resuspended in ice‐cold homogenization buffer (STE: 27% [w/w] sucrose, 50 mmol·L^−1^ Tris–HCl, pH 7.5 at room temperature, 5 mmol·L^−1^ EDTA) and homogenized with an Ultra‐Turrax homogenizer. The homogenate was pelleted at 300 × *g* for 5 min at 4°C, and the supernatant was centrifuged at 27,000 × *g* for 20 min at 4°C. The pellet was resuspended in ice‐cold 50 mmol·L^−1^ Tris–HCl, pH 7.5 at room temperature and 1 mmol·L^−1^ EDTA using a Dounce glass–glass homogenizer. It was centrifuged at 27,000 × *g* for 20 min at 4°C, and the pellet was resuspended again and homogenized.

#### 2.5.2 Binding assays using membranes

Binding reactions were performed in a 50‐μl volume that contained 0.5 μg of membranes, binding buffer (50 mmol·L^−1^ Tris–HCl, pH 7.5 at room temperature, and 0.1 mmol·L^−1^ EDTA, 5 mmol·L^−1^ MgCl_2_ and 0.1% BSA), 50 pmol·L^−1 125^I‐ANP and the indicated concentrations of NP and/or compounds or DMSO in a 96‐well format. Competition binding in the presence of 1 mmol·L^−1^ ATP was performed similarly, but with the addition of an ATP‐regenerating system to all wells (20 mmol·L^−1^ creatine phosphate, 0.2 mg·ml^−1^ creatine phosphokinase and 40 U·ml^−1^ myokinase final). The reactions were incubated for 2.5 h at room temperature before membranes were harvested onto Millipore harvest plates that had been pre‐soaked in 1% PEI through the use of a Packard Cell Harvester. Then the membranes were washed four times with cold 50 mmol·L^−1^ potassium phosphate buffer (pH 7.4 at room temperature). The filter plates were dried, and 20 μl MicroScint™‐O cocktail scintillation fluid (PerkinElmer) was added to each well before the plate was counted in a Packard TopCount Scintillation Counter (Packard Instrument Co.).

#### 2.5.3 Whole cell binding assays

This protocol was adapted from Dickey et al. (2009). QBIHEK293A cells that expressed GC‐A were seeded equally into 24‐well plates that had been coated with poly‐L‐lysine and grown to 90% confluency. The following day, cells were pretreated with DMEM that had been supplemented with 0.2% BSA for 1–2 h at 37°C. For the competition binding assays, cells were incubated in binding buffer (DMEM supplemented with 1% BSA) with 50 pmol·L^−1 125^I‐ANP and the indicated concentrations of NP and/or compounds or DMSO. For the saturation binding assays, cells were incubated in the same binding buffer with varying concentrations of ^125^I‐ANP and 0.1% DMSO (control) or 10 μmol·L^−1^ of the compound. For non‐specific binding, 1 μmol·L^−1^ ANP was added to the cells. Cells were incubated at 4°C for 1 h. After incubation, cells were washed twice with ice‐cold phosphate‐buffered saline (PBS) (pH 7.4 at room temperature), and the cells were harvested in 500 μl 1 mol·L^−1^ NaOH. The solution was transferred to scintillation vials, and radioactivity was counted using Ultima Gold XR scintillation cocktail (PerkinElmer) in a liquid scintillation counter (Tri‐Carb 2300 TR, Packard). Data were expressed as the percentage of maximum ^125^I‐ANP bound.

### 2.6 Phosphodiesterase activity assay

Cells were homogenized in ice‐cold 20 mmol·L^−1^ Tris–HCl (pH 7.5 at room temperature) that contained 1 mmol·L^−1^ EDTA, 1 mmol·L^−1^ dithiotreithol, 0.2 mmol·L^−1^ phenylmethylsulfonyl fluoride (PMSF), 0.1 mmol·L^−1^ sodium orthovanadate (Na_3_VO_4_), 1 mmol·L^−1^ benzamidine, 20 μg·ml^−1^ leupeptin and 10 μg·ml^−1^ aprotinin (trypsin inhibitor). The cGMP phosphodiesterase (PDE) activity was then measured by a modified two‐step procedure (Marchmont & Houslay, 1980), with modifications as previously described (Afzal et al., 2011).

### 2.7 Substrate‐velocity assay

GC assays were performed at 37°C in a reaction mixture that contained 25 mmol·L^−1^ HEPES (pH 7.4), 50 mmol·L^−1^ NaCl, 0.1% BSA, 0.5 mmol·L^−1^ IBMX, 1 mmol·L^−1^ EDTA, 5 mmol·L^−1^ phosphocreatine, creatine kinase (0.1 mg·ml^−1^), 0.5 mmol·L^−1^ microcystin and 5 mmol·L^−1^ MgCl_2_. The concentrations of GTP used are indicated in the figures. Reactions were initiated by adding 20 ml of crude membranes from GC‐A‐expressing HEK293T cells that contained 10–18 mg of protein suspended in phosphatase inhibitor buffer to 80 ml of the reaction mixture. Reactions were terminated in 110 mmol·L^−1^ ZnOAc and 110 mmol·L^−1^ Na_2_CO_3_ and purified on acidified alumina after elution in 3 ml of 200 mmol·L^−1^ ammonium formate to separate ATP and GTP from cGMP (Edmund et al., 2019). Concentrations of cGMP were determined by the performance of enzyme‐linked immunosorbent assays (ELISA) according to the manufacturer's instructions (ENZO Lifesciences).

### 2.8 Isolation of rat aortic rings and measurements of vascular reactivity

All animal care and experimental procedures complied with the UK Animals (Scientific Procedures) Act of 1986 and had approval from a local Animal Welfare and Ethical Review Body (P56EF988B). Animal studies are reported in compliance with the ARRIVE guidelines (Percie du Sert et al., 2020) and with the recommendations made by the *British Journal of Pharmacology* (Lilley et al., 2020). In all animal studies, male Wistar rats were used. Wistar is a commonly used rat strain and to reduce experimental variation and thereby the number of animals needed (reduction according to 3R), all experiments were done in male rats. Male Wistar rats (6 to 8 weeks old) were killed by CO_2_ asphyxiation. The thoracic aorta of each was dissected, and rings (~4 mm length) were mounted in organ baths that contained physiological salt solution (PSS: 119 mmol·L^−1^ NaCl, 4.7 mmol·L^−1^ KCl, 2.5 mmol·L^−1^ CaCl_2_, 1.2 mmol·L^−1^ MgSO_4_, 25 mmol·L^−1^ NaHCO_3_, 1.2 mmol·L^−1^ KH_2_PO_4_, and 5.5 mmol·L^−1^ glucose), which was maintained at 37°C and gassed with 5% CO_2_ in O_2_. Changes in isometric tension were measured in the tissues under a basal tension of 1 g. The viability of the tissue was assessed by exposure to KCl (80 mmol·L^−1^). Then, the maximal contractile response was recorded by exposure to a single dose of the thromboxane receptor agonist 9,11‐dideoxy‐11α,9α‐epoxymethano‐prostaglandin F_2α_ (U46619; 1 μmol·L^−1^). Arteries were then treated with the nitric oxide synthase (NOS) inhibitor L‐N^G^‐nitroarginine methyl ester (300 μmol·L^−1^) to block the production of endogenous nitric oxide. The arteries were then pre‐contracted with an 80% maximal effective (EC_80_) concentration of U46619. Once a stable response had been achieved, cumulative concentration–response curves were constructed either with increasing concentrations of compounds #2 or #20, or with ANP in the absence or presence of 10 μmol·L^−1^ of compound #2 or compound #20. In the latter case, compounds were incubated for 30 min prior to the administration of ANP. The relaxation was expressed as the means ± SEM as a percentage of the U46619‐induced tone.

### 2.9 Cell‐based cAMP assay

The level of NPR‐C agonism was assessed by quantifying the inhibition of forskolin‐induced cyclic adenosine‐3′,5′‐monophosphate (cAMP) production in HeLa cells. Cells were grown to around 80% confluency in 12‐well plates, and compounds (30 μmol·L^−1^) or the specific NPR‐C agonist cANF^4–23^ (100 nmol·L^−1^) were added and left to equilibrate with the cells for 10 min before the addition of forskolin (10 μmol·L^−1^). In some cases, the NPR‐C antagonist osteocrin (100 nmol·L^−1^) was added 10 min before the compounds were added. The reaction was stopped 20 min after forskolin addition and cells were lysed in HCl (0.1 mol·L^−1^) and centrifuged (18,620 × *g*, 2 min, 4°C). Concentrations of cAMP were measured in the supernatants through use of an ELISA (Direct cAMP; Enzo Life Sciences) following the manufacturer's instructions, and the values were normalized according to the protein concentration.

### 2.10 Construction of chimeric GC‐A/B and mutated receptors

Site‐directed mutagenesis in GC‐A and GC‐B and construction of chimeric receptors were performed through use of the In‐Fusion HD plus cloning kit (Takara Bio Inc.). The primers used are listed in Table S2. All plasmid sequences were verified by Sanger sequencing (Eurofins Genomics, GmbH).

### 2.11 Measurement of levels of cGMP in isolated rat cardiac fibroblasts

Cardiac fibroblasts were isolated from male Wistar rat hearts. Animal care was conducted according to the Norwegian Animal Welfare Act (and approved by the Norwegian Animal Research Authority, FOTS ID 23286), which conforms with the Directive 2010/63/EU of the European Parliament and of the European Council of 22 September 2010 on the protection of animals used for scientific purposes. Animals were anaesthetised with 4% isoflurane. Anaesthesia was confirmed by abolished pain reflexes and the euthanization was done by cervical dislocations. The chest was opened and the heart extracted. Hearts were perfused at 37°C using a Langendorff set‐up with buffer A (24 mmol·L^−1^ NaHCO_3_, 0.6 mmol·L^−1^ MgSO_4_, 1 mmol·L^−1^ DL‐carnitine, 10 mmol·L^−1^ creatine, 20 mmol·L^−1^ taurine and 0.1% BSA in Joklik‐modified minimum essential medium [MEM] [one ampoule (11 g) Joklik‐MEM per litre; M0518, Sigma‐Aldrich]) at a constant 6.4 ml·min^−1^ flow, equilibrated with 5% CO_2_/95% O_2_. Collagenase Type‐II (90 U·ml^−1^ final) (Worthington Biochemical Corporation, 268 U·mg^−1^) was added after 6 min and CaCl_2_ (0.2 mmol·L^−1^ final) after 24 min. When the aortic valves had been digested (33–35 min), the ventricles were minced and gently shaken at 37°C for 10 min in Buffer A with collagenase Type‐II and 0.2 mmol·L^−1^ CaCl_2_ with continuous 5% CO_2_/95% O_2_ supply. The suspension was filtered (nylon mesh, 250 μm) and centrifuged at 30 × *g* for 3 min at room temperature. The supernatant was kept (Supernatant 1), and the pellet was resuspended in 15–20 ml of buffer B (Buffer A plus 1% BSA and 0.5 mmol·L^−1^ CaCl_2_). The suspension was centrifuged once more at 30 × *g* for 4 min at room temperature, and the supernatant was combined with Supernatant 1. The combined supernatant was centrifuged at 1000 × *g* for 5 min at room temperature. The cell pellet was resuspended in Buffer A plus 0.5 mmol·L^−1^ CaCl_2_, 1% penicillin–streptomycin and 10% FBS to appropriate volume and incubated at 37°C for 2 h before the medium was changed. The fibroblasts were grown in DMEM (Gibco®, ThermoFischer Scientific) supplemented with 10% FBS, 100 U·ml^−1^ penicillin, 0.1 mg·ml^−1^ streptomycin and 0.25 mg·ml^−1^ amphotericin B and plated to six‐well plates in passage 2. The medium was changed to a serum‐free medium 24 h before experiments were conducted. Cells were pre‐incubated with compound #20 for 20 min and stimulated for 20 min with agonist as indicated. The experiment was stopped by the addition of 5% trichloroacetic acid (TCA), and the concentration of cGMP was measured using the cGMP enzyme immuno assay (EIA) kit (Cayman Chemical Company).

### 2.12 Data and statistical analysis

Statistical significance was determined using GraphPad Prism with either one‐way ANOVA, two‐way ANOVA or *t* test, as indicated. Bonferroni correction was applied for multiple comparisons, where appropriate. *P* < 0.05 was considered statistically significant. Statistical significance was only determined for group sizes of five or more, as recommended (Curtis et al., 2018). Exploratory data with n < 5 were included for illustration as appropriate, but not subjected to statistical analysis. Group size is the number of independent values used for statistical analysis. Where appropriate, data were normalized as explained. Only obvious outliers were removed before analysis. The data and statistical analysis comply with the recommendations of the *British Journal of Pharmacology* on experimental design and analysis in pharmacology (Curtis et al., 2018).

### 2.13 Materials

^125^I‐ANP was purchased from Phoenix Pharmaceuticals (Burlingame, CA, USA). NPs were obtained from GenScript (Leiden, Netherlands) and Sigma‐Aldrich (St. Louis, MO, USA) and proBNP from HyTest (Turku, Finland). Anti‐cGMP antibody was obtained from PerkinElmer (Waltham, MA, USA) and GenScript. Biotinylated cGMP was bought from PerkinElmer and BIOLOG (Bremen, Germany). Compounds #2 and #20 were obtained from MolPort (Riga, Latvia) and Mercachem (Nijmegen, Netherlands). Compound #20 was also synthesized by Drug Discovery Laboratory, AS (Oslo, Norway).

### 2.14 Nomenclature of targets and ligands

Key protein targets and ligands in this article are hyperlinked to corresponding entries in the IUPHAR/BPS Guide to PHARMACOLOGY (http://www.guidetopharmacology.org) and are permanently archived in the Concise Guide to PHARMACOLOGY 2021/22 (Alexander, Fabbro et al., 2021; Alexander, Kelly et al., 2021)

## 3 RESULTS

### 3.1 Identification of small molecule GC‐A modulators

**FIGURE 1:**
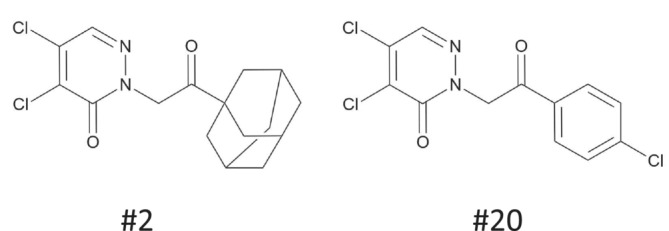
Molecular structures of compound #2, IUPAC name: 2‐[2‐(1‐adamantyl)‐2‐oxoethyl]‐4,5‐dichloropyridazin‐3‐one, and of compound #20, IUPAC name: 4,5‐dichloro‐2‐[2‐(4‐chlorophenyl)‐2‐oxoethyl]‐2,3‐dihydropyridazin‐3‐one.

**FIGURE 2:**
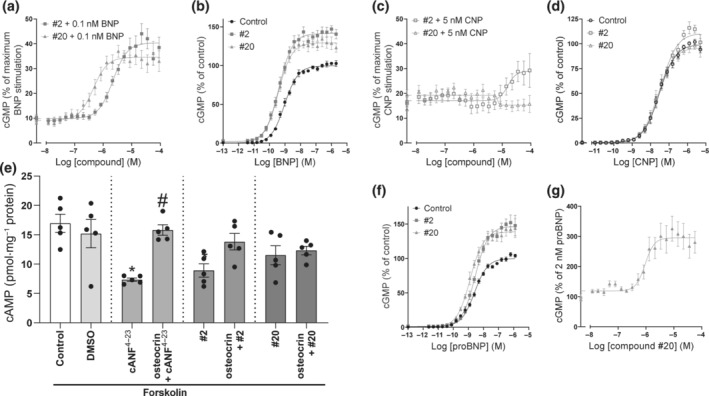
Compound #20 enhanced BNP‐ and unprocessed BNP‐induced GC‐A (a, b, f, g), but not CNP‐induced GC‐B activity (c, d) nor NPR‐C activity (e). (a, c) Concentration–response curves for compounds #2 and #20 in the presence of an EC_10_ of NP as indicated. The production of cGMP was normalized to the maximum NP‐mediated cGMP production in each experiment (n = 7 in a, n = 5 in c). Concentration–response curves for BNP (b) and CNP (d) in the absence or presence of 10 μmol·L^−1^ of compound #2 or #20 in cells that expressed GC‐A (n = 6) and GC‐B (n = 6), respectively. (e) Forskolin‐mediated cAMP production (control) was inhibited by NPR‐C activation in HeLa cells. Addition of the selective NPR‐C agonist cANF^4–23^ (100 nmol·L^−1^) as a positive control reduced cAMP accumulation, and this effect was reversed by the addition of the NPR‐C antagonist osteocrin (100 nmol·L^−1^) prior to stimulation. To investigate whether our compounds modulated NPR‐C activity, 30 μmol·L^−1^ of compounds was added prior to forskolin (10 μmol·L^−1^) stimulation, and osteocrin was added to investigate whether this reversed the effect (n = 5). (f) Concentration–response curves for proBNP in absence or presence of 10 μmol·L^−1^ compound #2 or #20 in GC‐A‐expressing cells (n = 4). (g) Concentration–response curve for compound #20 in the presence of 2 nmol·L^−1^ proBNP. The production of cGMP was normalized to proBNP alone (100%). (n = 6). Data shown are means ± SEM (a‐d, f, g) with individual values (e ). *P < 0.05, significantly different from control, ^#^ *P* < 0.05, significantly different from cANF^4–23^; one‐way ANOVA.

Activators of GC‐A were identified with high throughput screening. The assay was evaluated and validated based on Z′ values (Zhang et al., 1999). Z′ values were considered satisfactory for a cell‐based assay (0.44 ± 0.19). The cut‐off for hit compounds was set to >30% stimulation, and these compounds were re‐screened in triplicate and counter‐screened using non‐transfected QBIHEK293A cells. About 100 compounds were identified as hits and re‐screened in triplicate. Only compound #2 from the Enamine library (Figure 1) was verified as a hit. We tested about 100 analogues of compound #2 for activity towards GC‐A in a hit‐to‐lead process (Table S1). Compound #20 was identified as the most potent compound; it had 5‐ to 10‐fold higher potency than compound #2 (EC_50_ values of 508 ± 67 nmol·L^−1^ for compound #20; 3300 ± 800 nmol·L^−1^ for compound #2) (Figures 1 and 2a). The concentration–response curves for compounds were obtained in the presence of 0.1 nmol·L^−1^ human BNP, which generated 10% of maximum cGMP production (EC_10_). Compounds #2 and #20 showed similar efficacies and increased the cGMP production to 45% ± 5% and 35% ± 3% of the maximum BNP‐mediated cGMP production, respectively.

None of the compounds increased cGMP production in the absence of BNP, but both compounds #2 and #20 enhanced the effects of BNP (Figure 2b). These two compounds increased the potency of BNP (EC_50_: 1.0 ± 0.4 nmol·L^−1^) by 2.4 ± 0.5‐fold (#2) and 2.8 ± 0.4‐fold (#20) and increased the maximum BNP‐mediated cGMP production by 42% ± 5% and 30% ± 6%, respectively.

### 3.2 Compound #20 showed selectivity towards GC‐A

To determine selectivity towards GC‐A, compounds were tested for activity towards GC‐B and NPR‐C. Compound #2 showed some activity towards GC‐B at high concentrations, while compound #20 did not (Figure 2c). Neither of the compounds modulated the potency of CNP or increased the maximum level of CNP‐mediated cGMP production (Figure 2d).

Compounds were tested towards NPR‐C by measuring NPR‐C‐induced inhibition of cAMP production (Figure 2e). The presence of compound #20 did not reduce cAMP production and thus did not activate NPR‐C, while the presence of compound #2 reduced cAMP production and thus activated NPR‐C. This reduction was reversed when the NPR‐C antagonist osteocrin was added.

To rule out the possibility that the enhancement of GC‐A‐induced production of cGMP by our compounds was due to effects on PDEs, cGMP‐PDE activity was measured in homogenates from GC‐A‐expressing cells in the presence of our compounds. Neither of our compounds inhibited PDE activity alone or in the presence of IBMX (Figure S1).

### 3.3 Compounds modulated the effects of unprocessed BNP

Secretion of unprocessed BNP is increased in heart failure and may be of clinical significance (Costello‐Boerrigter et al., 2013). The precursor of BNP, proBNP, activates GC‐A with lower potency than BNP (Dickey & Potter, 2011b). We investigated whether compound #2 or #20 could modulate proBNP‐mediated production of cGMP. When increasing concentrations of proBNP were co‐incubated with 10 μmol·L^−1^ of compounds, compounds #2 and #20 increased cGMP production by 43% ± 10% and 42% ± 9%, respectively, but did not change the EC_50_ for proBNP (Figure 2f). In the presence of a low concentration of proBNP, compound #20 increased cGMP production in a concentration‐dependent manner by 177% ± 41% and had an EC_50_ of 1.1 ± 0.2 μmol·L^−1^ (Figure 2g).

### 3.4 Compound #20 increased BNP‐mediated cGMP production in cardiac fibroblasts

**FIGURE 3:**
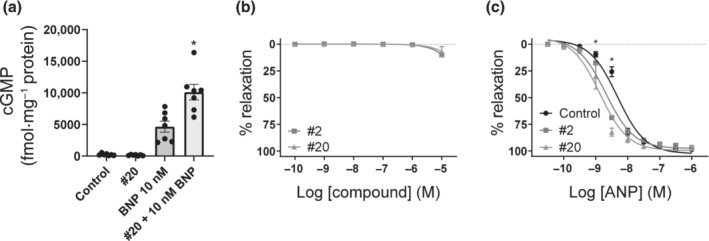
Compounds increased levels of BNP‐mediated cGMP in rat cardiac fibroblasts and modulated the vasorelaxant activity of ANP in isolated rat aorta. (a) Levels of cGMP in rat cardiac fibroblasts at basal levels (control), 10 μmol·L^−1^ #20, 10 nmol·L^−1^ BNP and pretreatment with 10 μmol·L^−1^ #20 followed by BNP stimulation (n = 7 rats). (b, c) Concentration–response of compounds #2 and #20 (n = 3 rats) (b) and concentration–response of ANP in the absence or presence of 10 μmol·L^−1^ compound #2 or #20 (n = 6–7 rats) (c) in isolated rat aorta pre‐contracted with U46619 and in the presence of L‐NAME. The relaxation is expressed as the means±SEM as a percentage of the U46619‐induced tone. Data shown are means ± SEM (b, c) with individual values (a). *P < 0.05, significantly different from BNP (a) or Control (c); two‐way ANOVA with Bonferroni correction.

In contrast with cell lines that overexpressed GC‐A, cardiac fibroblasts endogenously express GC‐A and activation inhibits cardiac fibrosis (Potter et al., 2006). We wanted to investigate the effects of our compounds in a system that contained physiological expression levels of GC‐A and stimulated isolated rat cardiac fibroblasts with BNP in the presence of DMSO or compound #20. The presence of compound #20 alone did not increase cGMP concentrations, but doubled the cGMP production (2.2 ± 1.2‐fold) in the presence of BNP, compared with the results of stimulation with BNP alone (Figure 3a).

### 3.5 Enhanced potency of ANP‐mediated vasorelaxation

Vasodilation contributes to the hypotensive properties of GC‐A activation and can be modelled ex vivo in rings of isolated rat aorta. To investigate the modulating effects of compounds #2 and #20, aortic rings that had been pre‐contracted with U46619 were stimulated with increasing concentrations of these compounds (Figure 3b) or with increasing concentrations of ANP in the presence or absence of 10 μmol·L^−1^ compound #2 or compound #20 (Figure 3c). Compounds did not modulate vasorelaxation alone but reduced the EC_50_ of ANP from 4.7 ± 0.4 nmol·L^−1^ to 2.2 ± 0.2 nmol·L^−1^ (#2) and to 1.6 ± 0.3 nmol·L^−1^ (#20).

### 3.6 Compound #20 increased the overall binding in whole cells

**FIGURE 4:**
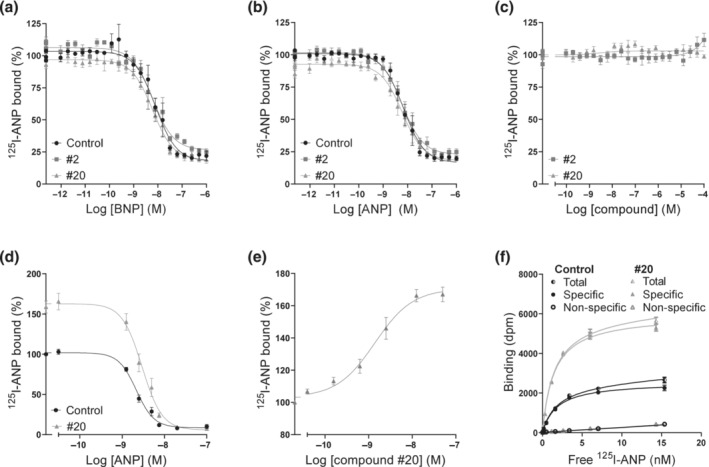
Compounds modulate binding, but not affinity, only in whole cells. (a–c) Competition binding curves using membranes from GC‐A‐expressing cells with increasing concentrations of BNP (n = 4) (a) and ANP (n = 7) (b) with 50 pmol·L^−1 125^I‐ANP in the absence or presence of 10 μmol·L^−1^ compound #2 or #20 or with increasing concentrations of compounds #2 and #20 (n = 5) (c). (d–f) Binding curves using whole cells. Competition binding curves with increasing concentrations of ANP in the absence or presence of 10 μmol·L^−1^ compound #20 (n = 5) (d) and increasing concentrations of compound #20 (n = 4) (e). Data points are means ± SEM. (f) Saturation binding analysis of ^125^I‐ANP in the presence (non‐specific binding) or absence of 1 μmol·L^−1^ ANP (total binding) and in the absence or presence of 10 μmol·L^−1^ compound #20. Specific binding was determined by subtracting non‐specific binding from total. Data points are means ± SEM of triplicates from one representative assay of four assays performed in total.

Binding assays that involved membranes from GC‐A‐expressing QBIHEK293A cells and ^125^I‐ANP were first performed to investigate effects on binding. Both ANP and BNP were able to displace ^125^I‐ANP from the receptor. However, there was no change in their binding or affinity in the presence of compound #2 or compound #20 (Figure 4a,b). The compounds did not displace ^125^I‐ANP or affect binding of ^125^I‐ANP in concentrations up to 100 μmol·L^−1^ (Figure 4c). However, when whole cells were used, compound #20 increased the overall binding of ANP by 60% ± 8% and slightly increased the IC_50_ from 2.3 ± 0.3 nmol·L^−1^ to 3.0 ± 0.4 nmol·L^−1^ (Figure 4d). Increasing concentrations of compound #20 increased the binding of ^125^I‐ANP by 71% ± 5% with an EC_50_ of 1.6 ± 0.4 nmol·L^−1^ (Figure 4e). In saturation binding assays, compound #20 did not change the affinity of ^125^I‐ANP, but increased the maximal binding 2.4 ± 0.1‐fold (Figure 4f).

### 3.7 Compounds did not interfere with the allosteric binding sites for ATP on GC‐A

**FIGURE 5:**
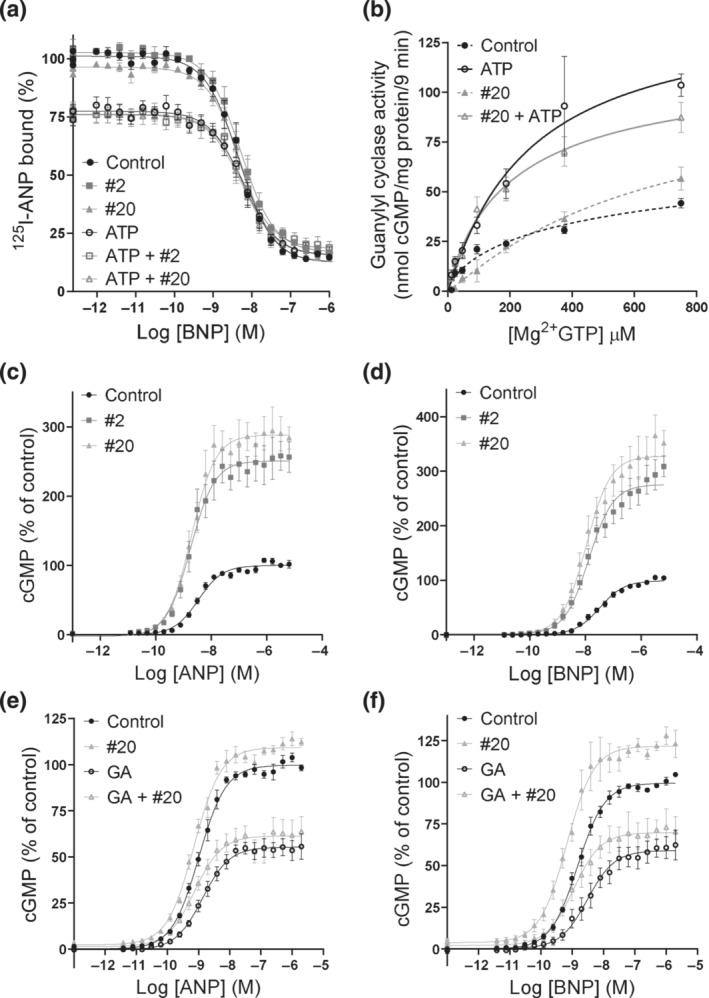
Compounds did not modulate the effects of ATP and are not dependent on phosphorylation state or HSP90 activity. (a) Competition binding curves with increasing concentrations of cold BNP and 50 pmol·L^−1 125^I‐ANP in membranes from GC‐A‐expressing cells with or without 1 mmol·L^−1^ ATP or compound #2 or #20 (n = 4). (b) Substrate‐velocity assay using membranes from HEK293T cells that expressed GC‐A. The curves represent GC activity in the presence of 1 μmol·L^−1^ ANP with or without 1 mmol·L^−1^ ATP and/or compound #20 and the indicated Mg^2+^GTP concentrations (n = 3). (c, d) Concentration–response curves for stimulation by ANP (n = 7) (c) or BNP (n = 5) (d) of cGMP production by the phosphomimetic mutant GC‐A^7E^ in the presence or absence of 10 μmol·L^−1^ compound #2 or #20. (e, f) ANP‐stimulated (n = 6) (e) and BNP‐stimulated (n = 5) (f) cGMP production in cells that expressed GC‐A in the presence or absence of 10 μmol·L^−1^ compound #20 and the chaperone HSP90 inhibitor geldanamycin (GA; 10 μmol·L^−1^). Data shown are means ± SEM.

Researchers have suggested that allosteric binding sites for ATP are present in the KHD and in the guanylyl cyclase domain. ATP binding to the KHD reduced the affinity of NPs for GC‐A (Jewett et al., 1993). The diuretic drug amiloride has been shown to antagonize this effect of ATP by binding to the same site and increasing the affinity of ANP (De Léan, 1986; Jewett et al., 1993). We wanted to explore whether compound #2 or #20 could antagonize the effect of ATP on the binding of ANP. In a competition binding assay with increasing concentrations of BNP, the presence of ATP reduced the binding of ^125^I‐ANP, but co‐incubation with the compounds #2 or #20, had no additional effects on binding (Figure 5a).

Previous studies have determined that ATP binds the pseudosymmetrical allosteric binding site in the catalytic domain of GC‐A, and that this binding decreases the K_m_ of GTP from >1 mmol·L^−1^ to physiological concentrations of approximately 100 μmol·L^−1^ (Antos & Potter, 2007; Robinson & Potter, 2012). Importantly, in the absence of ATP, GTP binds to the allosteric site, which results in positive cooperativity. Here, we investigated the possibility that compound #20 activated GC‐A by binding to the ATP allosteric site in the GC domain. However, in the absence of ATP, the presence of compound #20 did not change the K_m_ or cooperativity of the enzyme, which was inconsistent with the proposal that the compound bound to and activated this allosteric site (Figure 5b).

### 3.8 The effect of compound #20 is independent of phosphorylation of GC‐A

Phosphorylation of multiple residues in the juxtamembrane domain and the KHD is required for activation of GC‐A. In human GC‐A, there are seven phosphorylation sites (Yoder et al., 2010), and substitution of these sites with glutamate in GC‐A^7E^ to mimic the negative charge of phosphate also mimics the phosphorylated and active form of GC‐A (Otto et al., 2017). By using GC‐A^7E^, we investigated whether changes in phosphorylation contributed to the ability of compounds #20 and #2 to increase the activity of GC‐A (Figure 5c,d). The presence of either compound increased the level of ANP‐ or BNP‐mediated cGMP further in GC‐A^7E^ by 151% ± 23% (#2) and 183 ± 26% (#20) for ANP and 170% ± 25% (#2) and 221% ± 33% (#20) for BNP. The compounds also decreased the EC_50_ for both ANP (1.8 ± 0.2‐fold [#2] and 1.6 ± 0.2‐fold [#20]) and BNP (2.9 ± 0.6‐fold [#2] and 3.9 ± 1.1‐fold [#20]).

### 3.9 No modulation of chaperone activity towards GC‐A

GC‐A can be modulated indirectly by proteins that facilitate the correct folding of the enzyme. It has been shown that heat shock protein 90 (HSP90) is a chaperone that interacts with GC‐A and that inhibition of HSP90 with geldanamycin reduced levels of ANP‐mediated cGMP production (Kumar et al., 2001). To investigate whether our compounds were involved in HSP90 folding and trafficking of GC‐A, we inhibited HSP90 and measured levels of ANP‐ and BNP‐mediated cGMP production with and without the presence of compound #20 (Figure 5e,f). As in the previous study, inhibition of HSP90 reduced ANP‐ and BNP‐mediated cGMP production by 45% ± 11% and 41% ± 12%, respectively. We did not observe a change in the potency of ANP or BNP. When we inhibited HSP90, compound #20 increased the NP‐mediated cGMP production and reduced the EC_50_ of ANP and BNP, effects similar to those observed in the absence of HSP90 inhibition. The presence of compound #20 increased the cGMP production by 7% ± 6% (ANP) and 11% ± 5% (BNP) and reduced the EC_50_ by 1.7 ± 0.5‐fold and 3.6 ± 1.6‐fold for ANP and BNP, respectively.

### 3.10 The effect of compound #20 follows the intracellular domain of GC‐A

**FIGURE 6:**
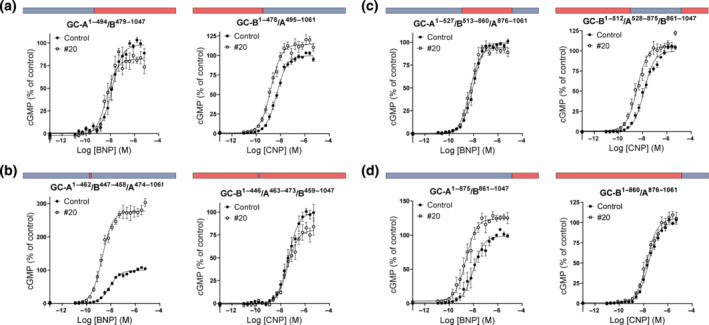
The activity of compound #20 followed the intracellular domain. Chimeric GC‐A (blue)/GC‐B (red) pairs stimulated with either increasing concentrations of BNP (GC‐A extracellular domain) or CNP (GC‐B extracellular domain) in the presence of 0.1% DMSO in control or 10 μmol·L^−1^ compound #20. The graphs show the activity of compound #20 after swapping the intracellular domain (left panel n = 6, right panel n = 8) (a), the juxtamembrane domain (left panel n = 4, right panel n = 4) (b), the kinase homology domain and the coiled‐coil domain (left panel n = 5, right panel n = 4) (c) and the guanylyl cyclase domain (left panel n = 4, right panel n = 4) (d) between GC‐A and GC‐B. Data shown are means ± SEM.

Human GC‐A and GC‐B are 57% identical in the extracellular domain and 78% identical in the intracellular domain. The dissimilarity in the extracellular domain is thought to explain their different affinities for NPs, while their intracellular domains are highly conserved, especially the guanylyl cyclase domain, which is 92% identical at the amino acid level. Compound #20 seemed to be highly selective towards GC‐A, with no effects towards GC‐B or NPR‐C. By making chimeric GC‐A/GC‐B in which domains, regions and amino acids had been swapped between the two receptors, we could investigate which part of GC‐A was essential for the activity of compound #20. The extracellular domain of GC‐A comprises approximately half of the receptor (amino acids 33–473), followed by a short 21‐amino acid transmembrane domain (amino acids 474–494) and an intracellular domain of 567 amino acids from 496 to 1061. When the intracellular domain of GC‐A was replaced with the intracellular domain of GC‐B (GC‐A^1–494^/B^479–1047^), compound #20 became inactive (Figure 6a). Conversely, for the analogous GC‐B^1–478^/A^495–1061^, the presence of compound #20 led to a change in the EC_50_ of CNP towards lower concentrations, but it did not increase the maximum CNP‐mediated cGMP production.

We were also interested in investigating a previously described allosteric binding site for the non‐selective antagonist HS‐142‐1 in the juxtamembrane domain in GC‐A and GC‐B (Poirier et al., 2002). When only the juxtamembrane domain was swapped between the two receptors, the selectivity of compound #20 towards GC‐A^1–462^/B^447–458^/A^474–1061^ was maintained (Figure 6b). Further, when the KHD and the CCD were swapped between the two receptors, the activity of compound #20 moved to GC‐B^1–512^/A^528–875^/B^861–1047^ and the activity towards GC‐A^1–527^/B^513–860^/A^876–1061^ was prevented (Figure 6c). Swapping only the KHDs or the CCDs between the two receptors yielded non‐functional GC‐B^1–512^/A^528–805^/B^787–1047^ and GC‐A^1–805^/B^787–860^/A^876–1061^, respectively. The activity of compound #20 was conserved between GC‐A^1–875^/B^861–1047^ and GC‐B^1–860^/A^876–1061^ where only the guanylyl cyclase domains were swapped between the two receptors (Figure 6d).

### 3.11 The effect of compound #20 depends on the amino acid residues at position 640 in GC‐A

**FIGURE 7:**
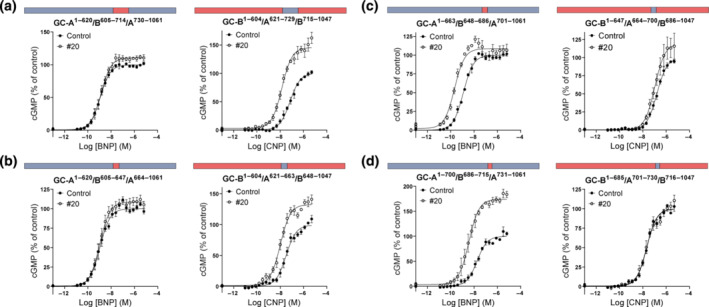
The activity of compound #20 depended on the presence of a small region in the kinase homology domain (KHD). Chimeric receptors with focus on the kinase homology domain and the dimerization domain showed that compound #20 was active only when the region of amino acid residues 621–661 was present. The activity moved to GC‐B when the corresponding amino acid residues were replaced with those of GC‐A^621–729^ (left panel n = 5, right panel n = 5) (a) or GC‐A^621–663^ (left panel n = 4, right panel n = 4) (b), but not GC‐A^664–700^ (left panel n = 3, right panel n = 4) (c) or GC‐A^701–730^ (left panel n = 4, right panel n = 4) (d). Data shown are means ± SEM.

**FIGURE 8:**
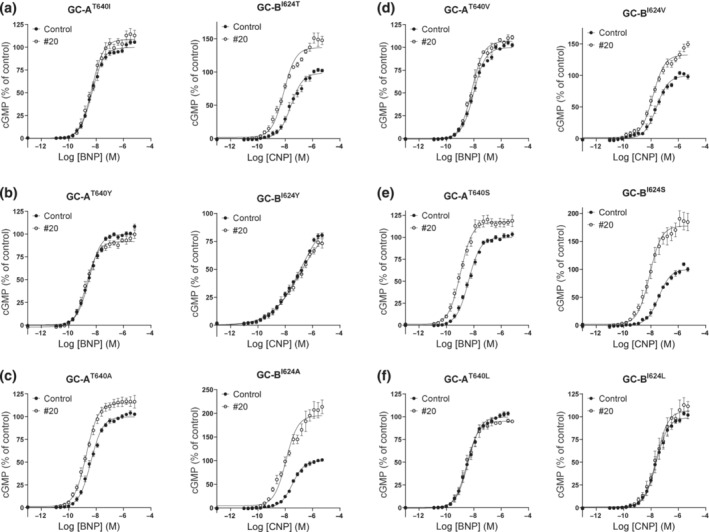
Only threonine‐640 mediates the effect of compound #20. (a) Swapping of nine non‐conserved amino acids in the region 621–663 between GC‐A and GC‐B revealed that the activity of compound #20 was only dependent on the presence of threonine‐640 in GC‐A (left panel n = 8, right panel n = 7). (b–f) Substitution of GC‐A^T640^ and the corresponding GC‐B^I624^ with the amino acids tyrosine (left panel n = 8, right panel n = 4), alanine (left panel n = 9, right panel n = 4), valine (left panel n = 4, right panel n = 5), serine (left panel n = 8, right panel n = 4) and leucine (left panel n = 4, right panel n = 4). Data shown are means ± SEM.

As the activity of compound #20 seemed to be determined by the KHD and/or CCD, we constructed and tested several chimeric receptors that were focused on this region from amino acids 528 to 875 in GC‐A (results summarized in Figure S2, and all graphs are shown in Figure S3). It became apparent that the activity of compound #20 followed the region from amino acids 621 to 729 in GC‐A, because most of the activity of compound #20 was lost for GC‐A^1–620^/B^605–714^/A^730–1061^ but gained in GC‐B^1–604^/A^621–729^/B^715–1047^ (Figure 7a). Further, the activity was lost in GC‐A^1–621^/B^605–647^/A^664–1061^ and present in GC‐B^1–604^/A^621–663^/B^648–1047^ (Figure 7b), whereas no change in activity was observed for GC‐A^1–663^/B^648–686^/A^701–1061^ and the corresponding GC‐B^1–647^/A^664–700^/B^686–1047^ (Figure 7c) or for GC‐A^1–700^/B^686–715^/A^731–1061^ and the corresponding GC‐B^1–685^/A^701–730^/B^716–1047^ (Figure 7d). The amino acid sequence from 621 to 663 in GC‐A is highly conserved in GC‐B and only nine amino acids differ between the two receptors. We constructed chimeric receptors that involved the switching of one or two amino acids between the two receptors (all graphs shown in Figure S4) and saw that the activity of compound #20 only followed amino acid Thr640 in GC‐A. Compound #20 became completely inactive towards GC‐A^T640I^ but it gained activity towards GC‐B^I624T^ (Figure 8a). By substituting Ile624 with Thr in GC‐B, compound #20 increased the maximum CNP‐mediated cGMP production by 35% ± 6% and decreased the EC_50_ for CNP 4.4 ± 0.7‐fold. However, the activity of compound #20 was not exclusively dependent on the presence of Thr. Compound #20 was also active when GC‐A^T640^ or GC‐B^I624^ were substituted with Ala and Ser (Figure 8c,e), but not when they were substituted with Tyr and Leu (Figure 8b,f). Compound #20 was not active in GC‐A^T640V^, but was active in GC‐B^I624V^ (8d). Substitution of Thr640 (GC‐A) or Ile624 (GC‐B) with glutamic acid or aspartic acid produced non‐functional receptors.

The structure of the intracellular domains of GC‐A or GC‐B has not yet been solved, but homology models of their KHDs have been published (Edmund et al., 2019). These models were based on conserved structure similarities between KHDs in GC‐A and GC‐B and protein kinases. In addition, the structures of the full‐length receptors have been predicted using deep‐learning system AlphaFold (Jumper et al., 2021). In both models, GC‐A^T640^ and GC‐B^I624^ are buried in an α‐helical region and are not readily accessible from the surface (Figure S5).

## 4 DISCUSSION

Here, we show that small, low MW compounds can increase BNP‐ or ANP‐mediated cGMP production and thereby enhance the efficacy of these peptides towards GC‐A. Importantly, one of our compounds (i.e., #20) did not affect GC‐B or NPR‐C activity and thus showed selectivity towards GC‐A. We further identified Thr640 in GC‐A as the amino acid essential for this effect.

### 4.1 Small molecule allosteric enhancers of GC‐A and possible clinical relevance

GC‐A is an attractive target for the treatment of cardiovascular diseases because its activation leads to vasodilation and has an overall cardiorenal protective effect. The current strategy is to use BNP, ANP and designer NPs, but their inherently short half‐life and poor bioavailability limit their potential uses. Another strategy is to increase their concentrations by inhibiting neprilysin and hence preventing their catabolism. However, neprilysin is not selective towards NPs. It also catabolizes angiotensin II and other hormones that counteract the effects of NPs.

In contrast with previous strategies, small molecular modulators uniquely enhance the effects of endogenous ANP and BNP towards GC‐A. Modulation of allosteric binding sites can have several advantages besides their lack of competition with NPs towards their binding site. Allosteric binding sites are thought to be less conserved than orthosteric sites, so there is an opportunity to use more selective compounds (Wenthur et al., 2014). We have shown that the activity and selectivity of compound #20 depend on the presence of one residue that differs between GC‐A and GC‐B. In addition, allosteric modulators can be safer than orthosteric agonists, as their effects are limited to both the presence and the concentration of the endogenous agonist. Too much GC‐A activation can lead to hypotension and was shown to be a safety concern when using BNP in treating heart failure (O'Connor et al., 2011).

### 4.2 Novel allosteric binding site on GC‐A

In our search for the allosteric binding site of our compounds, we started to investigate known allosteric binding sites in the extracellular and intracellular domains of GC‐A. Our GC‐A/B chimeric constructs excluded the possibility that the sought site was one of two known extracellular allosteric binding sites on GC‐A (Figure 6a,b). The first of these was a site for a chloride atom in the extracellular domain that regulates binding of ANP to GC‐A and has been suggested to be an allosteric modulator in the kidneys (Misono, 2000; Ogawa et al., 2010). The second was the putative allosteric binding site for the non‐selective GC‐A antagonist HS‐142‐1 in the juxtamembrane domain (Poirier et al., 2002). In the intracellular domain, the chimeric receptors also excluded the possibility that our compounds were bound to the allosteric binding sites that have been suggested for ATP in the KHD and GC domains (Antos & Potter, 2007; De Léan, 1986; Jewett et al., 1993; Robinson & Potter, 2012). Testing of more chimeric GC‐A/B receptors narrowed the search for the putative binding site to a region in the KHD of GC‐A and finally pinpointed a single amino acid. However, the activity of compound #20 at the chimeric receptors was not always similar to that at GC‐A. For some chimeric receptors, the presence of compound #20 affected only the EC_50_ or the maximum cGMP level but not both. This could reflect differences in receptor expression levels and receptor reserve in the transfected cells (Lohse et al., 1990). In a few chimeric pairs, compound #20 was active at both, but more at the chimeric receptors containing Thr640. The activity of compound #20 followed that of GC‐A^T640^ in all chimeric pairs, except for GC‐A^1–805^/B^787–1047^ and the corresponding GC‐B^1–786^/A^806–1061^, in which it reduced the BNP‐mediated cGMP production by GC‐A^1–805^/B^787–1047^. These deviating results might imply that the activity of compound #20 on these chimeric GC‐A/B receptors could be affected by intramolecular interactions between homologous regions of GC‐A and GC‐B. However, no amino acid substitution other than that of GC‐A^T640^ prevented the activity, and activity in GC‐B was restored by substitution of the analogous GC‐B^I624T^.

Our chimeric GC‐A/B receptors and point mutations do not provide definite evidence of a binding site. Mutations in one part of GC‐A or GC‐B can affect the function of the receptor elsewhere through intramolecular interactions. From the existing models of GC‐A and GC‐B, we see that both GC‐A^T640^ and GC‐B^I624^ are buried in an α‐helix (Figure S5) and may not be readily accessible for a small molecule to interact with. The binding site may be located at a different and more accessible site on the receptor, but the effect is mediated through GC‐A^T640^. Our mutagenesis studies do not suggest another binding site, but the location of GC‐A^T640^ challenges our findings. We also lack evidence of a direct interaction between compound #20 and GC‐A. This could be investigated further through resolution of the structure of GC‐A with compound #20 bound.

### 4.3 Mechanisms of action

Allosteric enhancers are compounds that enhance the affinity and/or efficacy of the orthosteric agonist while having no effect on their own (Neubig et al., 2003). In our concentration–response curves, we did not detect any effects of the compounds alone on cGMP production (Figure 2a,f), vasorelaxation (Figure 3b) or on basal GC‐A activity (Figure 3a). In contrast to the compound MCUF‐651 found by Sangaralingham et al. (2021), our binding experiments showed that #2 and #20 did not affect the affinities of ANP or BNP in either whole cells or membranes. However, compound #20 increased the overall binding in whole cells (Figure 4). Therefore, we explored the known mechanisms of action of allosteric modulators of GC‐A, which modulate cGMP production and the efficacy of NPs. The presence of ATP increases the efficacy of the catalytic guanylyl cyclase domain by decreasing K_m_ and increasing V_max_ (Robinson & Potter, 2011; Robinson & Potter, 2012)_,_ but it has also been shown to reduce the overall binding of ANP to GC‐A (De Léan, 1986; Jewett et al., 1993). Nevertheless, none of our compounds modulated the effects of ATP on binding and they did not change the efficacy of the enzyme in our GC assays with or without ATP present (Figure 5a,b). We also investigated known methods of indirect modulation of GC‐A and found that our compounds did not affect the effect of HSP90 or increase cGMP production by inhibiting PDEs. These findings indicate that our compounds allosterically enhance the efficacy of NPs through a novel, and yet unknown, mechanism of action. The increased binding of NPs due to compound #20 in whole cells indicates that #20 increases receptor density at the cell surface. Thus, one can speculate that compound #20 either interferes with internalization and degradation of the receptors or receptor recycling. However, further studies are required to investigate this.

To identify small molecules that activated GC‐A, we performed a high throughput screening. Through this process, only one compound was identified as a hit. Small molecular GC‐A agonists have been described previously (Iwaki, Nakamura, et al., 2017; Iwaki, Oyama, et al., 2017; Iwaki, Tanaka, et al., 2017), and a positive allosteric enhancer (i.e., MCUF‐651) has recently been reported (Sangaralingham et al., 2021). Of note, MCUF‐651 has a different chemical structure compared to our compounds, and its binding site remains unknown. Furthermore, MCUF‐651 seems to have a different mechanism of action. Indeed, unlike compound #20 and #2, MCUF‐651 affects the affinity of ANP. Importantly, while our compound #20 showed selectivity towards GC‐A and no effects upon the NPR‐C, such selectivity was not demonstrated for MCUF‐651, thus limiting its potential clinical use. Certainly, while the clinical relevance of this new and long‐awaited class of compounds is well recognized, more studies are warranted to better define the properties, the mechanisms of action and the clinical characteristics of these potential innovative drugs.

Our low hit rate and the fact that only a few low MW compounds are known to activate GC‐A could be due to the structure and large interface of the orthosteric binding site. The use of small molecules to mimic NP activation of GC‐A remains a challenge in the utilization of GC‐A as a potential drug target. Here, we provide the structure and activity of two allosteric enhancers of GC‐A and suggest a novel allosteric binding site. Both compounds could serve as tool compounds for further development and proof‐of‐concept of allosteric enhancement of GC‐A.

## AUTHOR CONTRIBUTIONS

All authors designed the research. Cristina Pérez‐Ternero, Jerid Robinson, Deborah M. Dickey, Adrian J. Hobbs, Lise Román Moltzau and Henriette Andresen performed research and analysed data. Henriette Andresen wrote the manuscript with input from all authors. All authors approved the final version.

## CONFLICT OF INTEREST STATEMENT

A.C., F.O.L., L.R.M. and H.A. are inventors named on a patent application related to allosteric enhancers of GC‐A and treatment of hypertension.

## DECLARATION OF TRANSPARENCY AND SCIENTIFIC RIGOUR

This Declaration acknowledges that this paper adheres to the principles for transparent reporting and scientific rigour of preclinical research as stated in the *BJP* guidelines for Design and Analysis, and Animal Experimentation, and as recommended by funding agencies, publishers and other organizations engaged with supporting research.

## Supporting information


**Figure S1.** Compounds did not inhibit PDE activity. cGMP‐PDE activity in GC‐A‐expressing cells treated with 0.1% DMSO (control) or 10 μM compound #2 or #20 in the presence or absence of the general PDE inhibitor IBMX. Data points are means ± SEM (n = 3–7).
**Figure S2**. Illustrative summary of the results from all tested chimeric GC‐A/B receptors. The effect of compound #20 versus control on BNP or CNP stimulation towards chimeric GC‐A/B. The effects of compound #20 are quantified as fold change in EC_50_ ± SEM and percentage change ± SEM in the maximal NP‐mediated cGMP production. Compound #20 increased (↑), decreased (↓) or had no effect (−) on the EC_50_ or cGMP production during testing of the illustrated chimeric GC‐A/B. Some chimeric receptors were not active (NA) in response to NP stimulation. Effects were analysed as difference between control and compound #20 and validated using *t* test. *P ≤ 0.05, The individual graphs are shown in Figure S2. TM, transmembrane domain; KHD, kinase homology domain; CCD, dimerization domain; GC, guanylyl cyclase domain
**Figure S3**. All chimeric GC‐A/B tested. Concentration–response curves for chimeric GC‐A/B pairs with the indicated concentrations of BNP (GC‐A extracellular domain) or CNP (GC‐B extracellular domain) stimulated with 0.1% DMSO (control) or 10 μM compound #20. Some chimeric receptors were not active (NA). Data points are means ± SEM (n: see Figure S2).
**Figure S4**. Mutations of non‐conserved amino acids reveal that the activity only occurred in the presence of GC‐A^T640^. Concentration–response curves for BNP and CNP and the effects of compound #20 towards GC mutations with single or dual amino acid swapping of non‐conserved amino acids. In the region 621–663 in GC‐A, only nine amino acids are nonconserved between GC‐A and GC‐B. Data points are means ± SEM (n = 3–5).
**Figure S5**. GC‐A^T640^ and GC‐B^I624^ are buried in an alpha helical region. Homology models (a) and predicted models (b) of kinase homology domain of GC‐A and GC‐B in which GC‐A^T640^ is in yellow and GC‐B^I624^ is in pink.

**Table S1.** Structures and activity screening (n = 1) of compounds obtained in in silico design and during hit‐to‐lead. Activities and results of compound #2 and #20 are shown in the main manuscript. The structures are here for comparison.
**Table S2**. a. Overview of primers used in constructing chimeric GC‐A/B. The pcDNA3.1(+) vector was linearized by restriction enzymes HindIII and XbaI and isolated from hGC‐A or hGC‐B pcDNA3.1(+) plasmids. Two or three DNA fragments were fused together with the linearized vector using the In‐Fusion HD Enzyme premix that recognize a 15–20 bp overlap in the ends of each fragment. This overlap were added to the PCR primers.

## Data Availability

The data that support the findings of this study are available from the corresponding author upon reasonable request. Some data may not be made available because of privacy or ethical restrictions.
