# Supplementary material for: Novel enhancers of guanylyl cyclase‐A activity acting via allosteric modulation
Source: Br J Pharmacol. 2023 Aug 29;180(24):3254–70. doi: 10.1111/bph.16203 (PMC10952227; doi:10.1111/bph.16203)
Supplement: Supplementary file 1 — Figure S1. Compounds did not inhibit PDE activity. cGMP‐PDE activity in GC‐A‐expressing cells treated with 0.1% DMSO (control) or 10 μM compound #2 or #20 in the presence or absence of the general PDE inhibitor IBMX. Data points are means ± SEM (n = 3–7). Figure S2. Illustrative summary of the results from all tested chimeric GC‐A/B receptors. The effect of compound #20 versus control on BNP or CNP stimulation towards chimeric GC‐A/B. The effects of compound #20 are quantified as fold change in EC50 ± SEM and percentage change ± SEM in the maximal NP‐mediated cGMP production. Compound #20 increased (↑), decreased (↓) or had no effect (−) on the EC50 or cGMP production during testing of the illustrated chimeric GC‐A/B. Some chimeric receptors were not active (NA) in response to NP stimulation. Effects were analysed as difference between control and compound #20 and validated using t test. *P ≤ 0.05, The individual graphs are shown in Figure S2. TM, transmembrane domain; KHD, kinase homology domain; CCD, dimerization domain; GC, guanylyl cyclase domain Figure S3. All chimeric GC‐A/B tested. Concentration–response curves for chimeric GC‐A/B pairs with the indicated concentrations of BNP (GC‐A extracellular domain) or CNP (GC‐B extracellular domain) stimulated with 0.1% DMSO (control) or 10 μM compound #20. Some chimeric receptors were not active (NA). Data points are means ± SEM (n: see Figure S2). Figure S4. Mutations of non‐conserved amino acids reveal that the activity only occurred in the presence of GC‐AT640. Concentration–response curves for BNP and CNP and the effects of compound #20 towards GC mutations with single or dual amino acid swapping of non‐conserved amino acids. In the region 621–663 in GC‐A, only nine amino acids are nonconserved between GC‐A and GC‐B. Data points are means ± SEM (n = 3–5). Figure S5. GC‐AT640 and GC‐BI624 are buried in an alpha helical region. Homology models (a) and predicted models (b) of kinase homology domain of GC‐A and GC‐B in [file BPH-180-3254-s001.pdf]

Supporting information Fig. S1

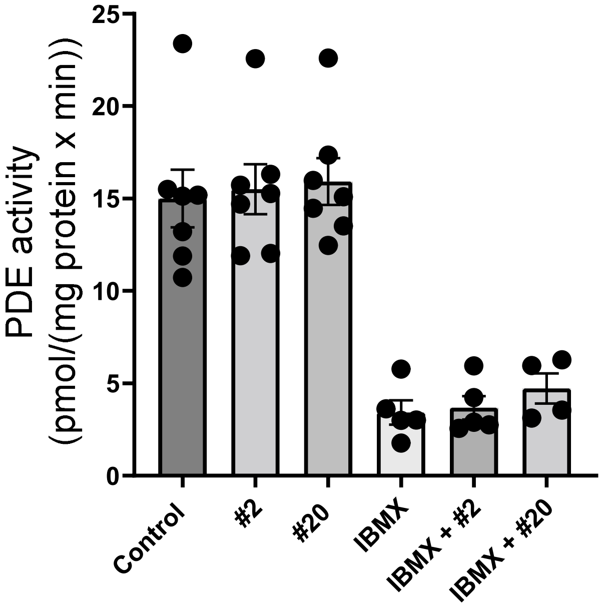

Supplementary Fig. S1. Compounds did not inhibit PDE activity. cGMP-PDE activity in GC-A-expressing cells treated with 0.1% DMSO (control) or 10 μM compound #2 or #20 in the presence or absence of the general PDE inhibitor IBMX. Data points are means±SEM (n=3-7).

Supporting information Fig. S2

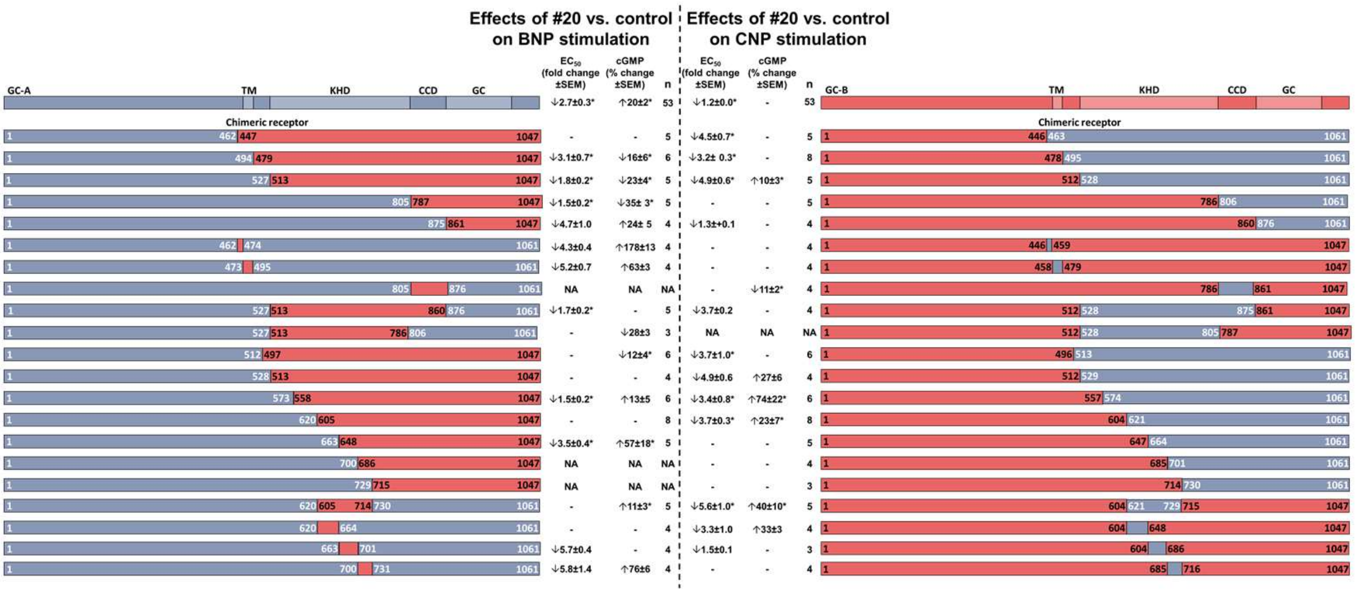

Supplementary Fig S2. Illustrative summary of the results from all tested chimeric GC-A/B receptors. The effect of compound #20 versus control on BNP or CNP stimulation towards chimeric GC-A/B. The effects of compound #20 are quantified as fold change in EC_{50}±SEM and percentage change±SEM in the maximal NP-mediated cGMP production. Compound #20 increased (↑), decreased (↓) or had no effect (-) on the EC_{50} or cGMP production during testing of the illustrated chimeric GC-A/B. Some chimeric receptors were not active (NA) in response to NP stimulation. Effects were analyzed as difference between control and compound #20 and validated using t-test. *$p \leq 0.05$, The individual graphs are shown in Supplementary Fig. S2. TM, transmembrane domain; KHD, kinase homology domain; CCD, dimerization domain; GC, guanylyl cyclase domain.

Supporting information Fig. S3

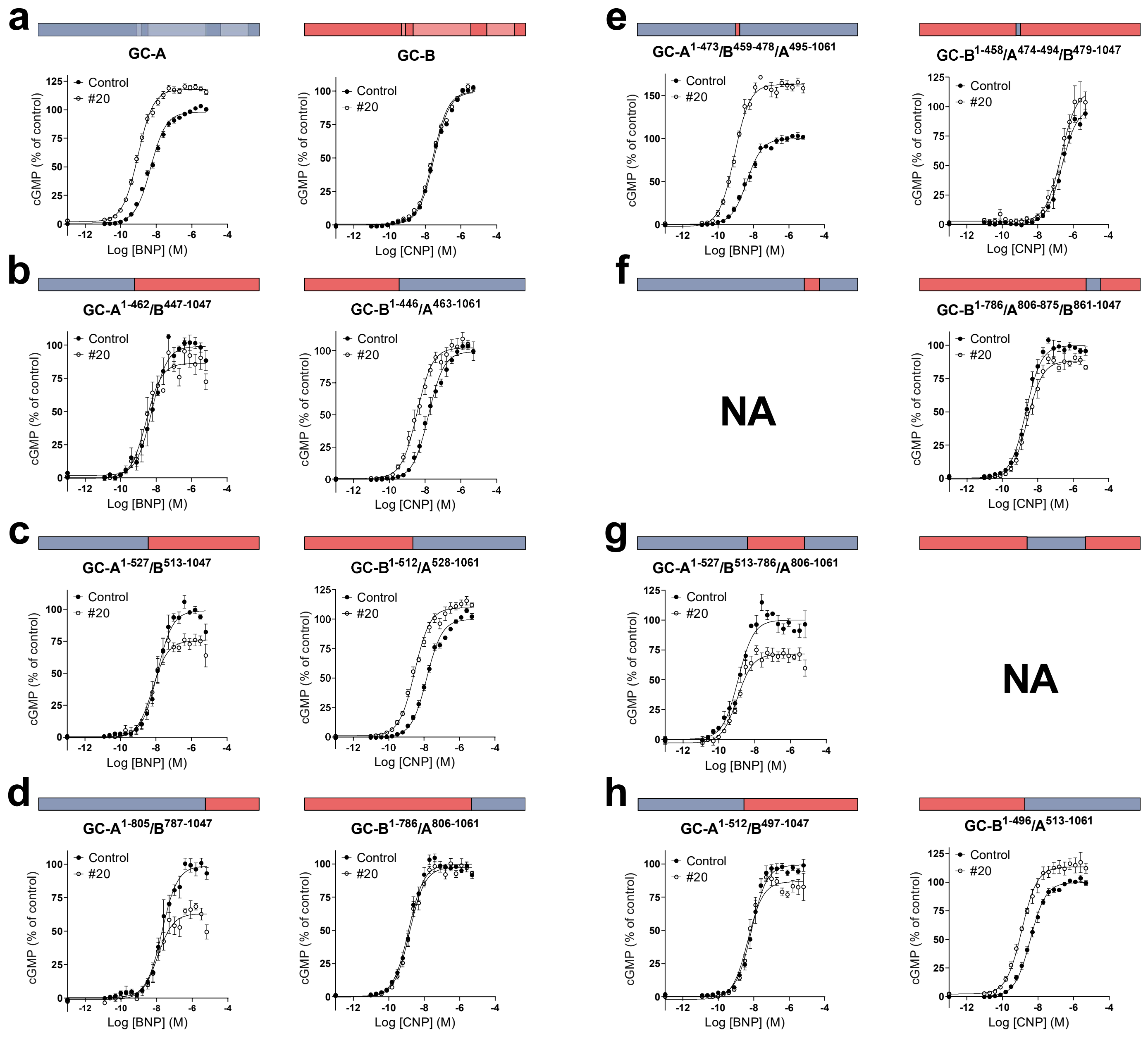

Supporting information Fig. S3 – cont.

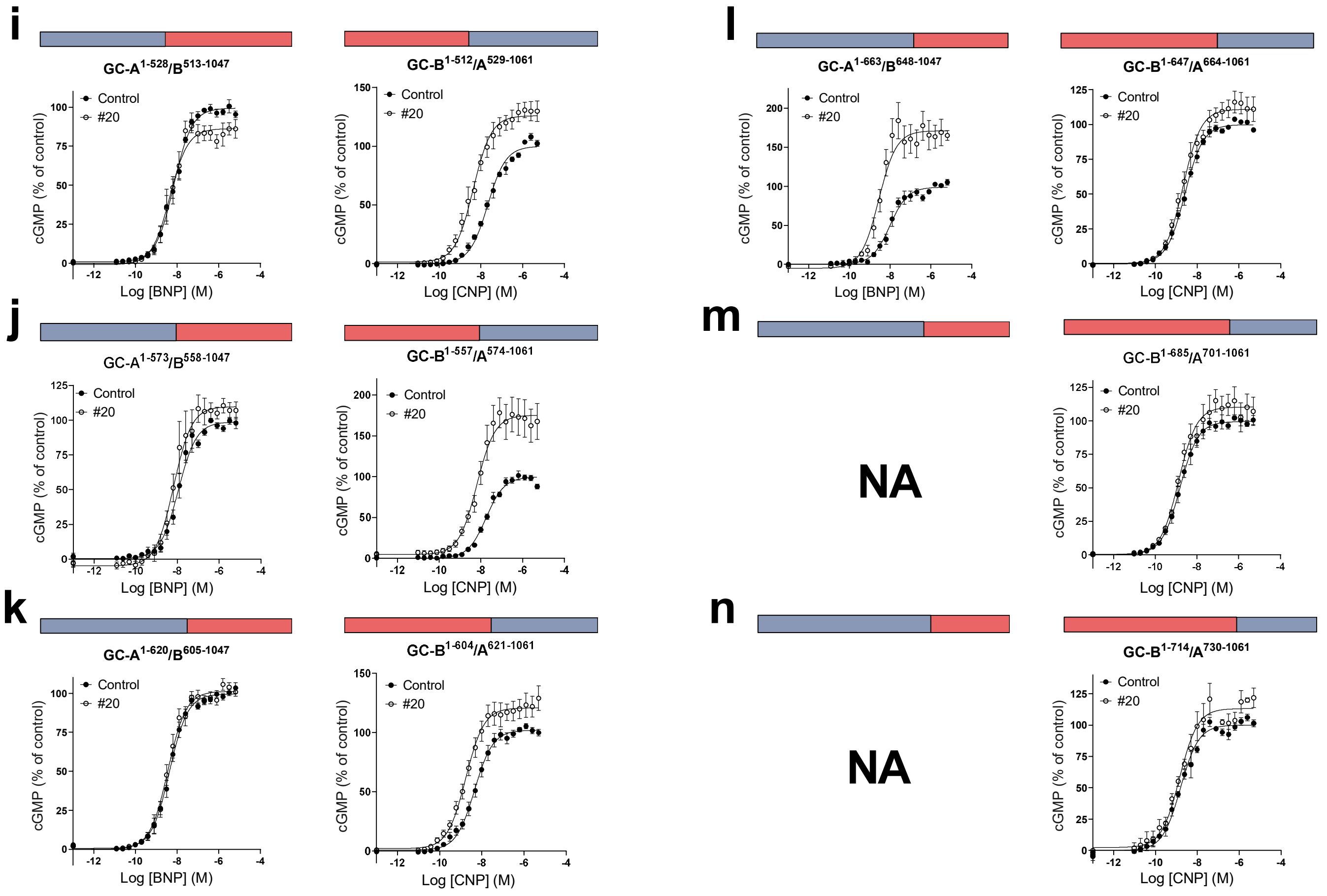

Supplementary Fig. S3. All chimeric GC-A/B tested. Concentration-response curves for chimeric GC-A/B pairs with the indicated concentrations of BNP (GC-A extracellular domain) or CNP (GC-B extracellular domain) stimulated with 0.1% DMSO (control) or 10 µM compound #20. Some chimeric receptors were not active (NA). Data points are means±SEM (n: see figure S2).

Supporting information Fig. S4

GC-A 621 QDILEN**E**SI**T**LDWMFRYSL**T**ND**I**VKGM**L**FLHN**GA**I**C**SHG**N**LKS 663
GC-B 605 QDILEN**D**SI**N**LDWMFRYSL**I**ND**L**VKGM**A**FLHN**SI**I**S**SHG**S**LKS 647

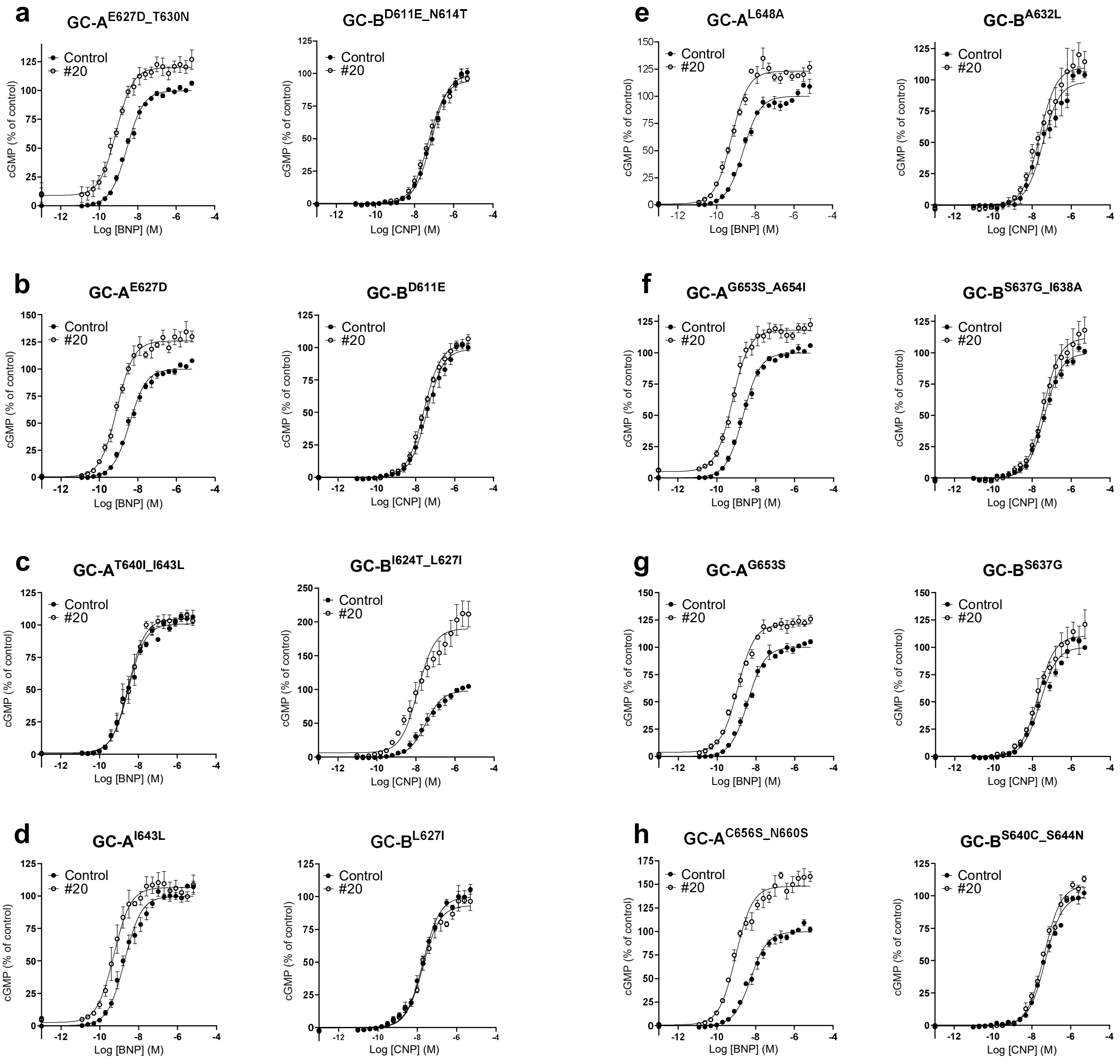

Supplementary Fig. S4. Mutations of non-conserved amino acids reveal that the activity only occurred in the presence of GC-A^{T640}. Concentration-response curves for BNP and CNP and the effects of compound #20 towards GC mutations with single or dual amino acid swapping of non-conserved amino acids. In the region 621-663 in GC-A, only nine amino acids are non-conserved between GC-A and GC-B. Data points are means±SEM (n=3-5).

Supporting information Fig. S5

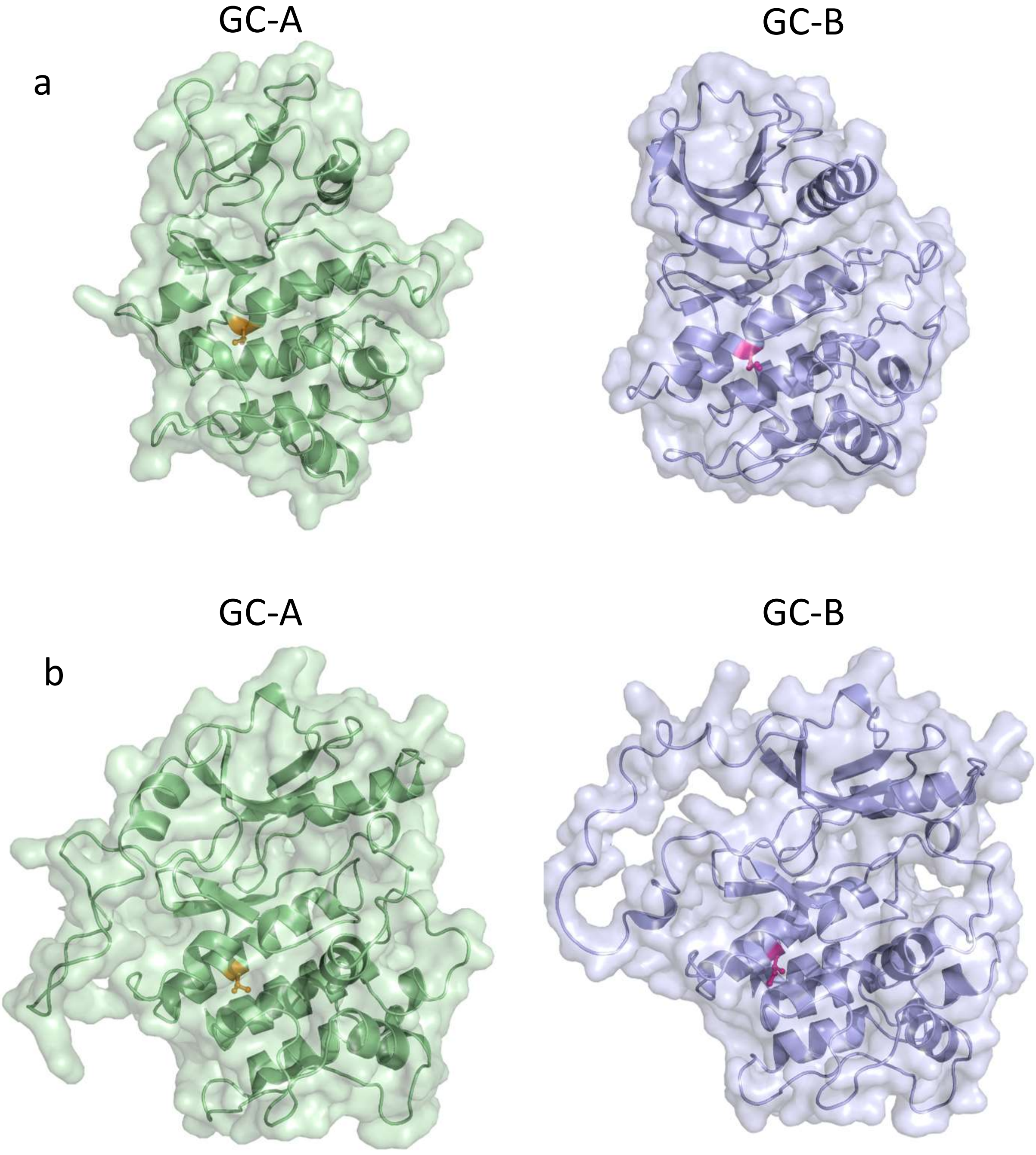

Supplementary Fig. S5. GC-A^{T640} and GC-B^{I624} are buried in an alpha helical region. Homology models (**a**) and predicted models (**b**) of kinase homology domain of GC-A and GC-B in which GC-A^{T640} is in yellow and GC-B^{I624} is in pink.
