# Supplementary material for: Novel enhancers of guanylyl cyclase‐A activity acting via allosteric modulation
Source: Br J Pharmacol. 2023 Aug 29;180(24):3254–70. doi: 10.1111/bph.16203 (PMC10952227; doi:10.1111/bph.16203)
Supplement: Supplementary file 2 — Table S1. Structures and activity screening (n = 1) of compounds obtained in in silico design and during hit‐to‐lead. Activities and results of compound #2 and #20 are shown in the main manuscript. The structures are here for comparison. Table S2. a. Overview of primers used in constructing chimeric GC‐A/B. The pcDNA3.1(+) vector was linearized by restriction enzymes HindIII and XbaI and isolated from hGC‐A or hGC‐B pcDNA3.1(+) plasmids. Two or three DNA fragments were fused together with the linearized vector using the In‐Fusion HD Enzyme premix that recognize a 15–20 bp overlap in the ends of each fragment. This overlap were added to the PCR primers. [file BPH-180-3254-s002.pdf]

SUPPORTING INFORMATION

Supporting table I. Structures and activity screening (n=1) of compounds obtained in in silico design and during hit-to-lead.
Activities and results of compound #2 and #20 are shown in the main manuscript. The structures are here for comparison.

		Activity, % above control			Efficacy, %	$-\log EC_{50}$	BNP efficacy, % above control
		3 nM	40 nM	300 nM			
#1	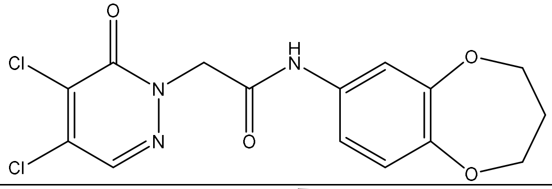	ND	ND	ND	NA	NA	30.5
#2	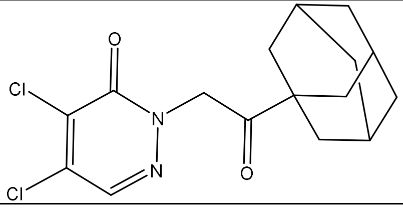						
#3	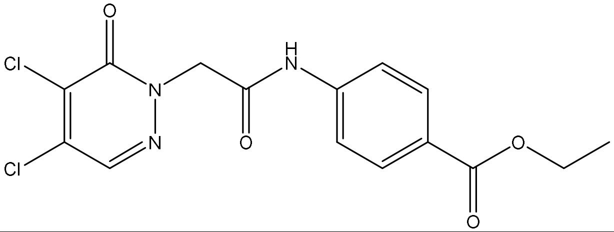	ND	ND	ND	NA	NA	34.4

#4	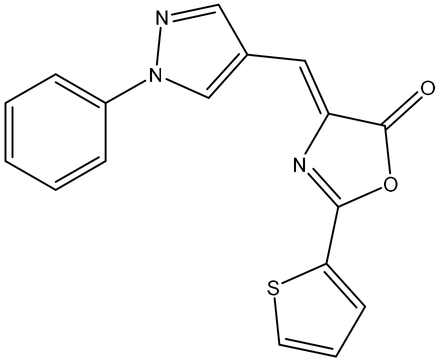	ND	ND	ND	NA	NA	27.0
#5	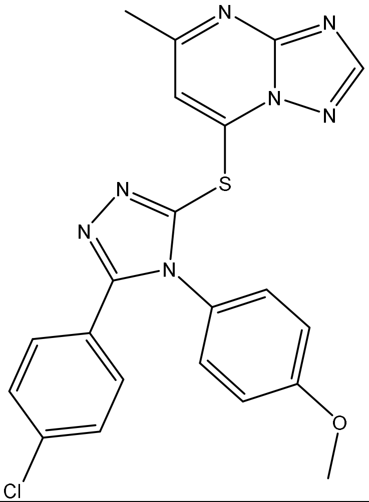	ND	ND	ND	NA	NA	6.3

#6	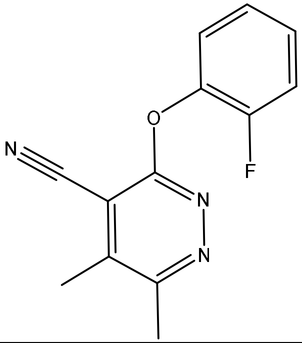	ND	ND	ND	NA	NA	2.9
#7	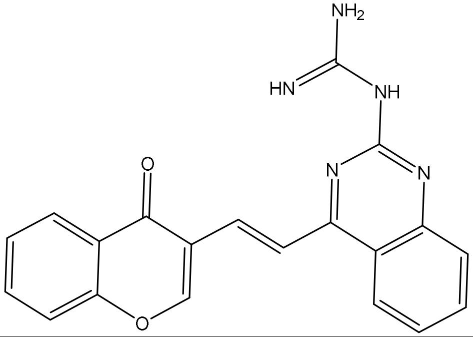	ND	ND	ND	NA	NA	-0.8
#8	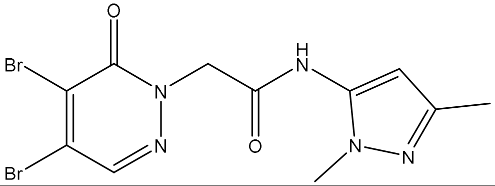	ND	ND	ND	NA	NA	16.7
#9	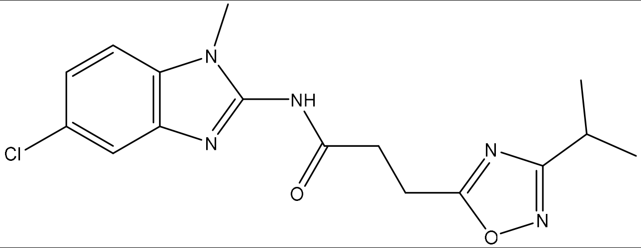	ND	ND	ND	NA	NA	9.7

#10	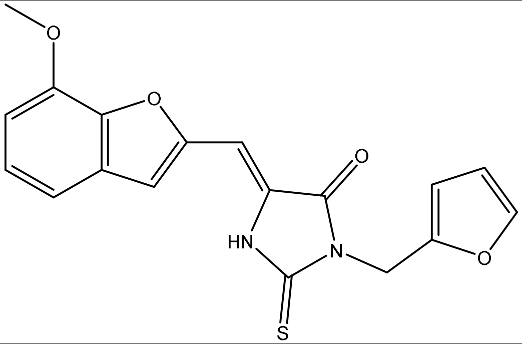	ND	ND	ND	NA	NA	-0.3
#11	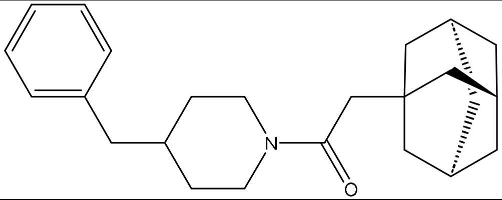	-9.9	-22.8	-15.2	ND	ND	ND
#12	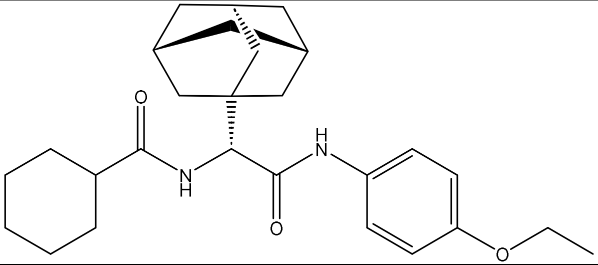	-20.5	-16.0	-13.2	ND	ND	ND
#13	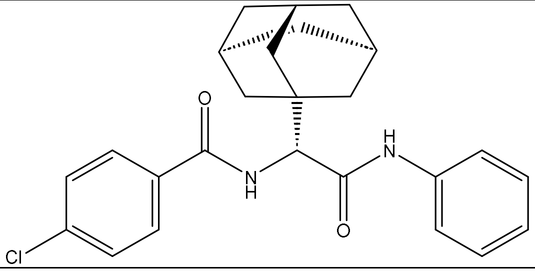	-18.1	-31.3	-36.5	ND	ND	ND

#14	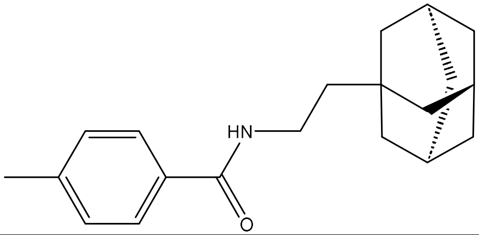	NA	-17.6	-7.9	ND	ND	ND
#15	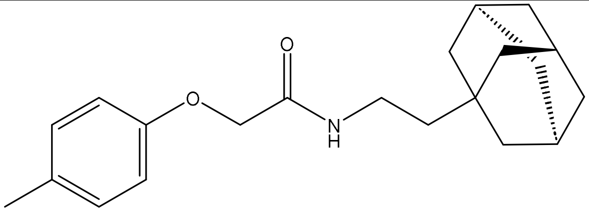	9.1	-9.7	-13.8	ND	ND	ND
#16	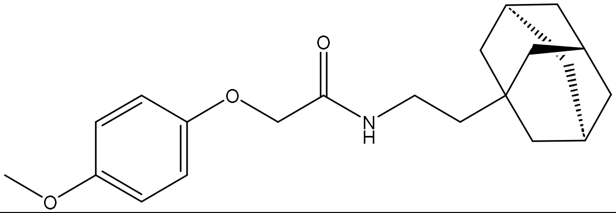	-4.7	-32.2	-37.5	ND	ND	ND
#17	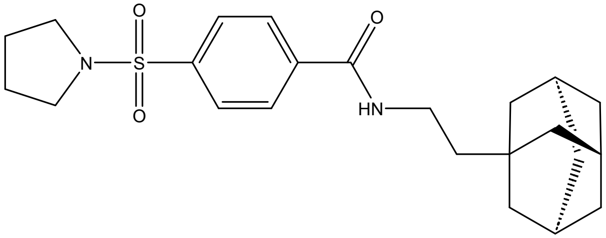	-5.8	-38.7	-34.5	ND	ND	ND

#18	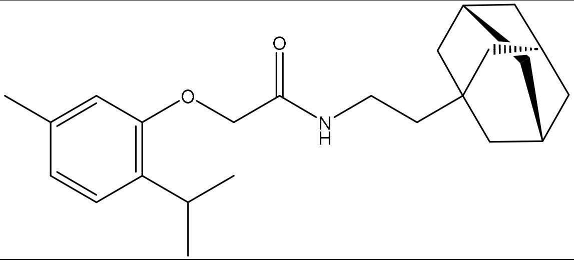	-7.6	-37.5	-32.8	ND	ND	ND
#19	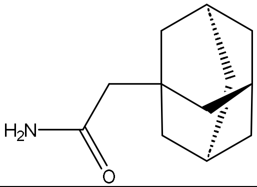	-9.3	-31.9	-39.4	ND	ND	ND
#20	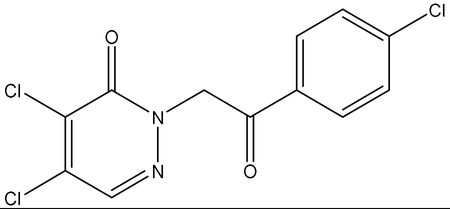						
#21	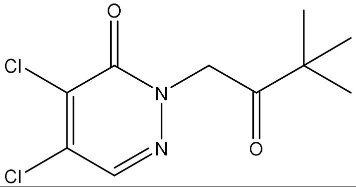	52.8	10.0	-6.9	38.5	4.85	ND
#22	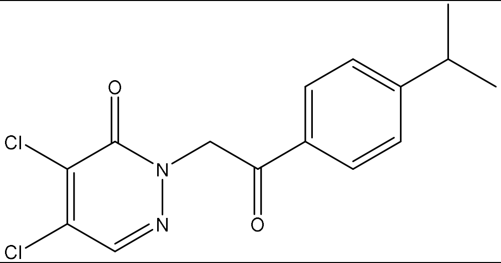	-1.5	-11.0	-10.9	ND	ND	ND

#23	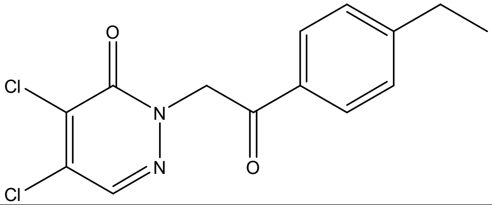	20.3	-7.8	-17.6	ND	ND	ND
#24	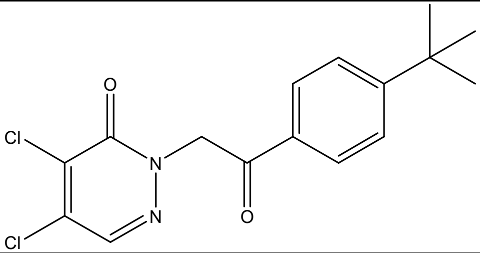	38.0	14.7	-2.8	36.4	5.35	-2.9
#25	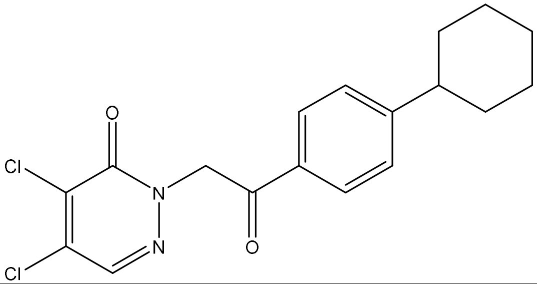	195.7	136.9	114.8	32.2	5.76	84.7
#26	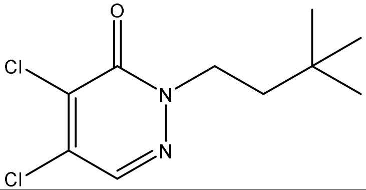	-12.8	-17.8	-12.2	ND	ND	ND

#27	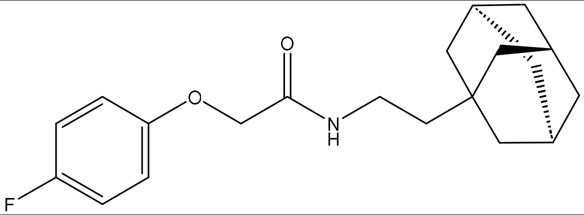	-24.7	-35.5	-35.9	ND	ND	ND
#28	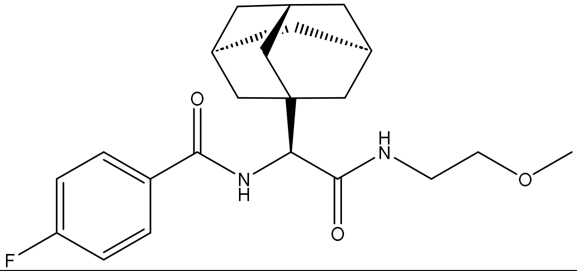	-30.5	-39.4	-35.4	ND	ND	ND
#29	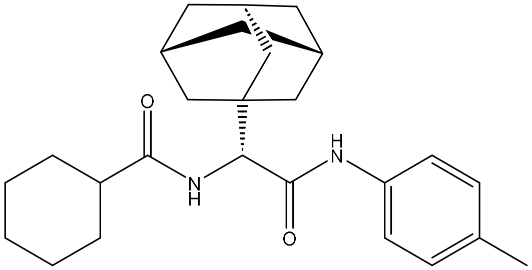	-31.2	-33.2	-33.5	ND	ND	ND
#30	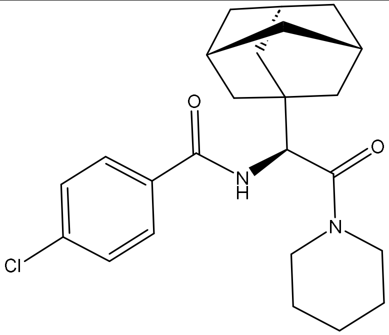	-11.4	-9.6	-9.2	ND	ND	ND

#31	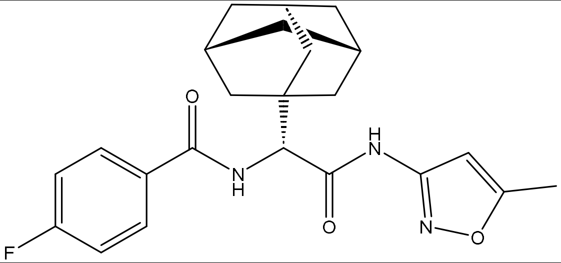	14.8	18.9	-0.4	ND	ND	ND
#32	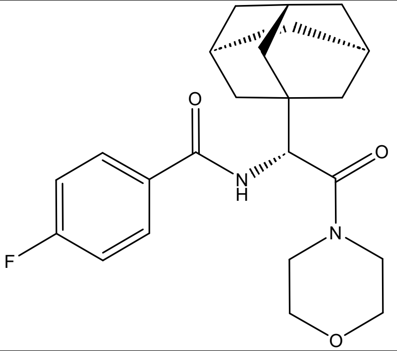	12.3	10.9	-5.3	ND	ND	ND
#33	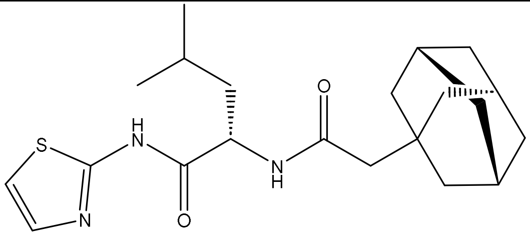	-2.5	7.1	-11.9	ND	ND	ND
#34	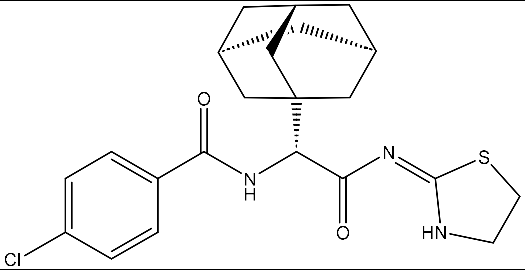	9.7	14.8	-6.8	ND	ND	ND

#35	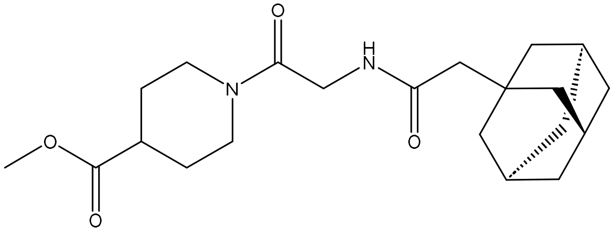	3.6	22.9	-9.0	ND	ND	ND
#36*	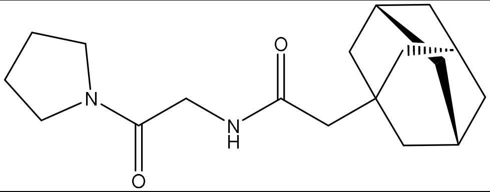	ND	ND	ND	ND	ND	ND
#37	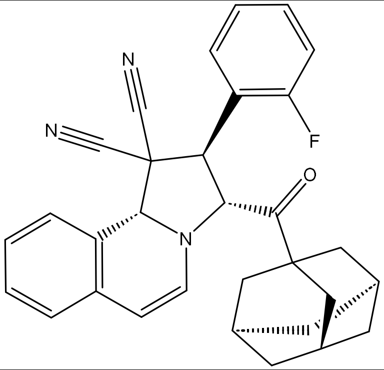	2.7	15.7	-20.3	ND	ND	ND
#38	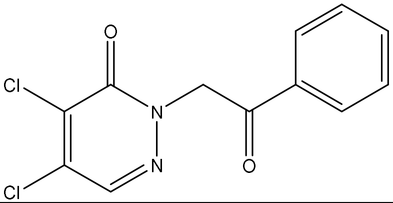	89.2	26.4	26.4	48.4	4.64	25.8

#39	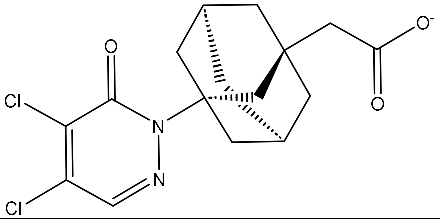	-5.7	6.9	-1.0	ND	ND	ND
#40*	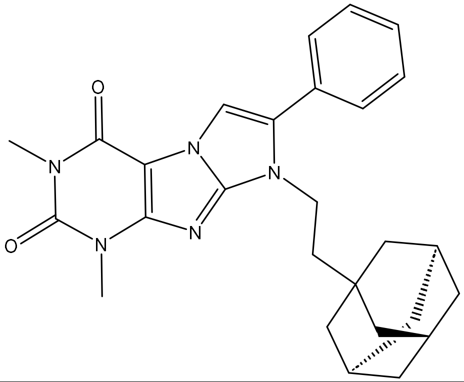	ND	ND	ND	ND	ND	ND
#41	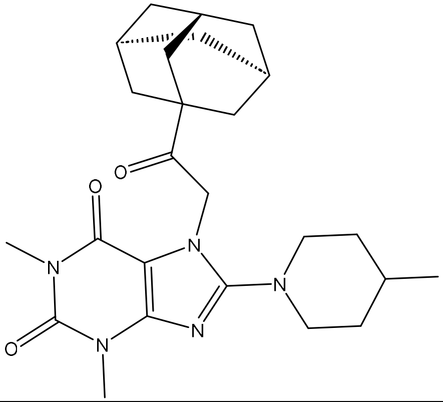	-35.9	-8.5	-14.5	ND	ND	ND

#42	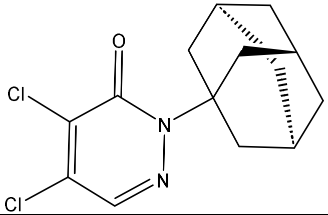	6.9	26.7	2.5	ND	ND	ND
#43	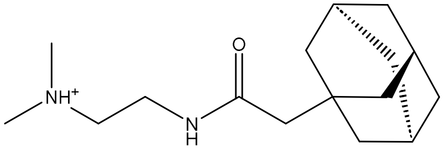	-7.2	-11.9	18.4	ND	ND	ND
#44	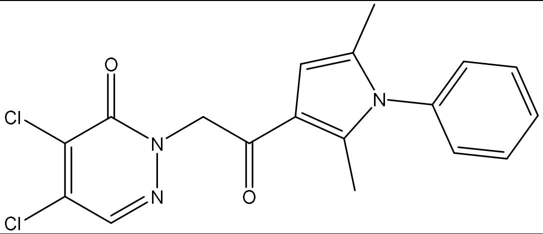	18.3	3.7	-21.8	ND	ND	ND
#45	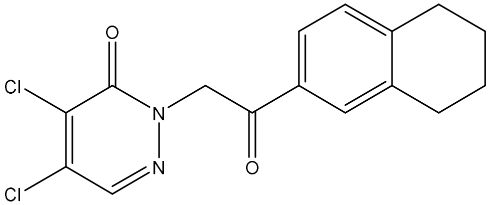	244.7	182.1	54.3	18.1	5.9	ND
#47	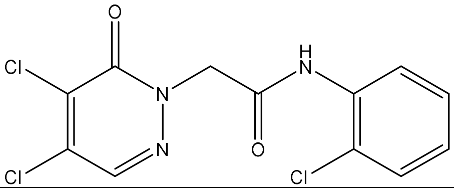	84.3	159.6	120.7	9.4	5.31	ND

#48	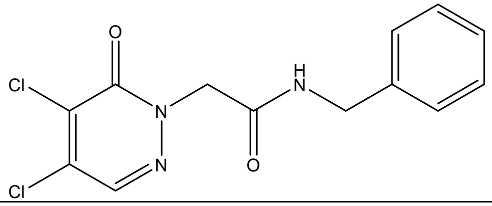	71.6	30.3	4.5	9.2	7.31	ND
#49	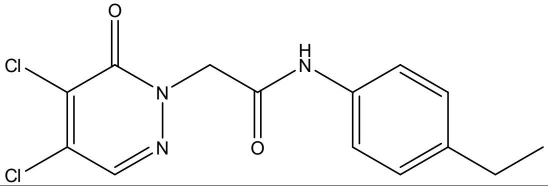	72.9	-3.1	-28.4	17.3	4.83	ND
#50	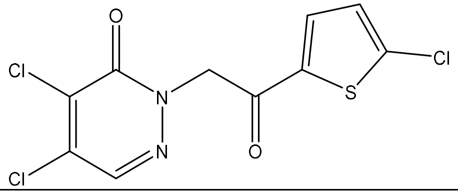	289.3	143.1	52.8	12.9	6.59	22.8
#51	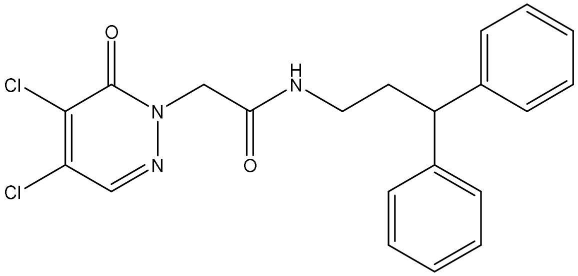	-6.2	-36.2	-45.5	ND	ND	ND

#52	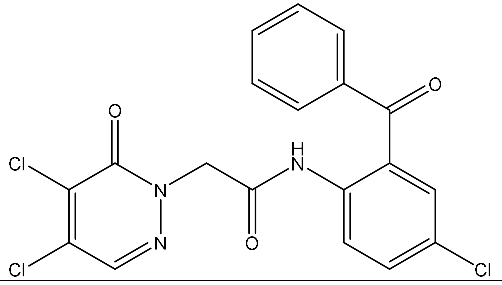	-69.3	-43.5	-43.0	ND	ND	ND
#53	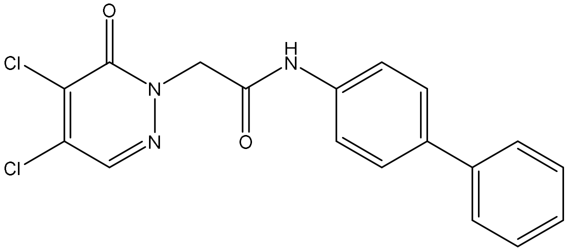	NA	-96.4	-95.6	ND	ND	ND
#54	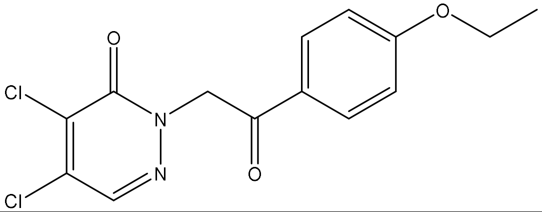	42.8	-4.0	-17.1	18.6	4.45	-10.6
#55	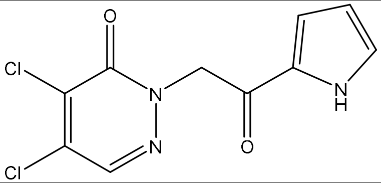	72.7	59.4	40.7	8.8	5.62	ND

#56	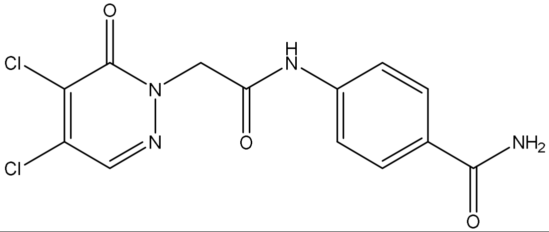	44.6	-16.1	-31.3	7.4	5.07	ND
#57	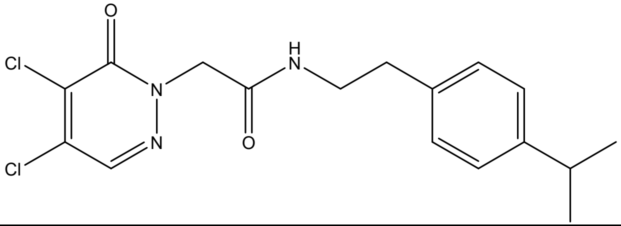	213.8	122.0	64.4	17.1	5.16	11.1
#58	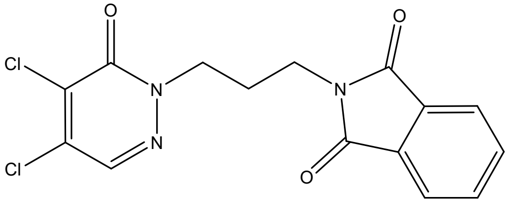	65.6	34.8	61.2	8.5	5.5	ND
#59	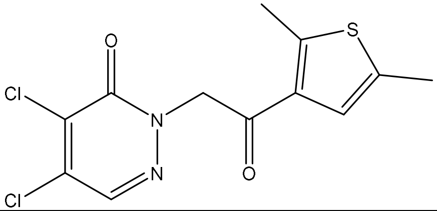	74.0	28.0	11.3	15.1	5.88	ND

#60	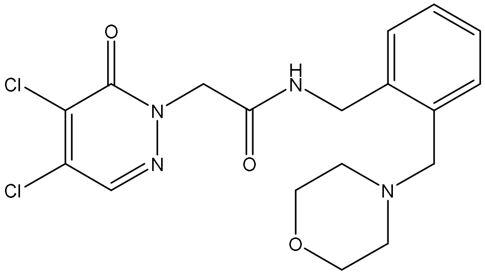	-14.9	-29.6	-12.2	ND	ND	ND
#61	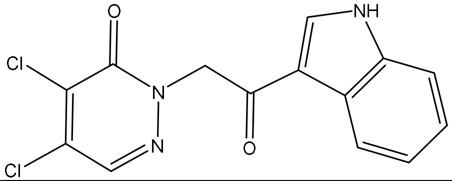	69.9	5.2	-1.8	21.2	4.62	1.3
#62	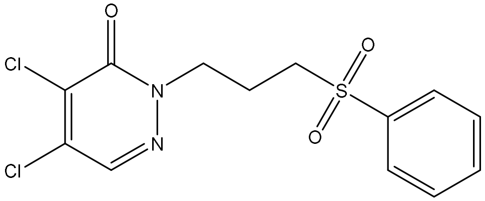	11.1	9.0	32.6	ND	ND	ND
#63	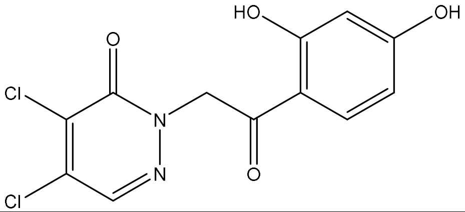	75.7	8.4	0.9	ND	ND	ND
#64	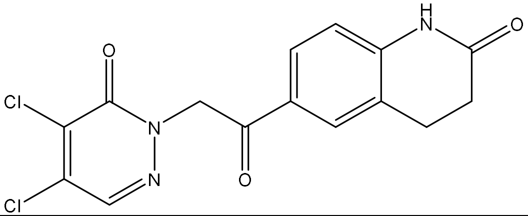	199.9	97.5	79.7	8.4	5.72	ND

#65	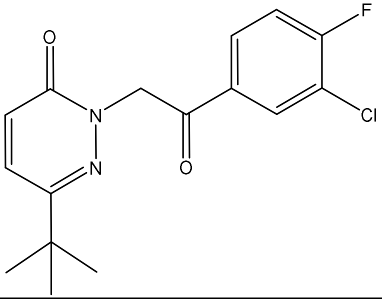	28.8	4.8	-12.7	ND	ND	ND
#66	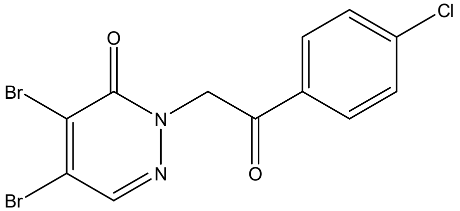	246.2	46.7	30.2	27.6	5.76	-8.7
#67	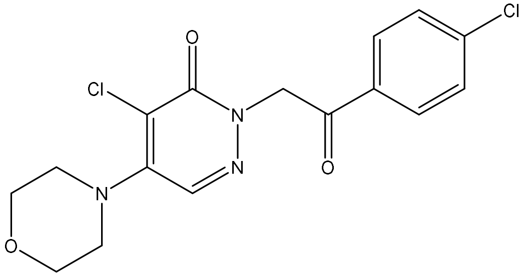	54.2	11.0	-3.3	ND	ND	ND
#68	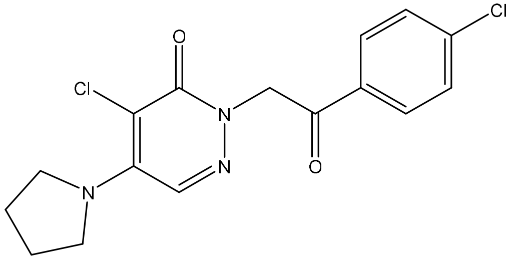	52.0	13.4	1.1	ND	ND	ND

#69	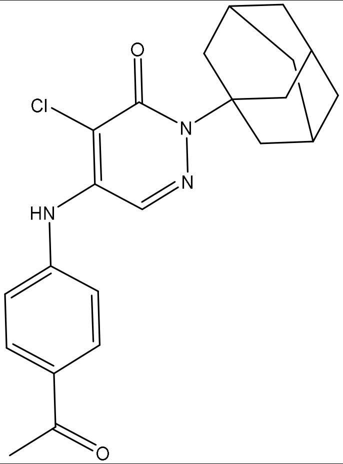	206.6	49.2	2.1	33.9	5.86	10.2
#70	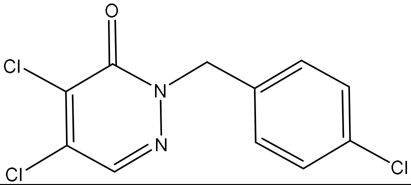	147.4	65.7	75.4	1.4	4.39	55.0
#71	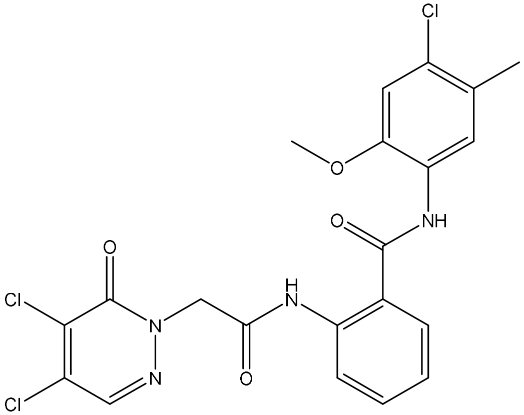	127.9	104.1	25.3	21.3	5.77	52.6

#72	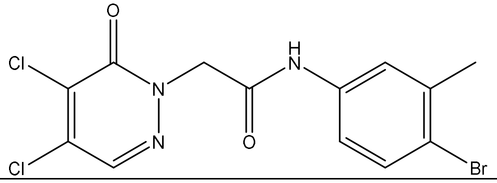	116.4	53.2	19.0	0.7	4.42	173.1
#73	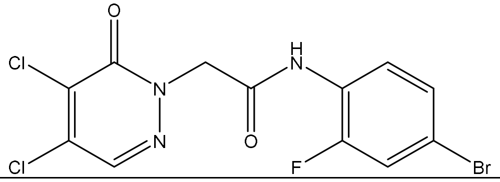	163.6	94.0	35.5	36.3	4.98	95.6
#74	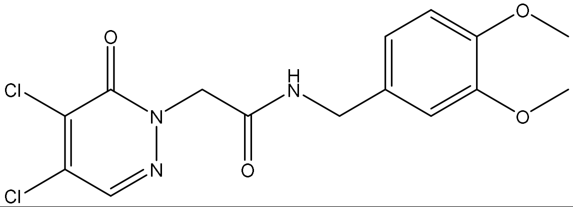	169.8	107.3	25.8	52.7	6.16	44.8
#75	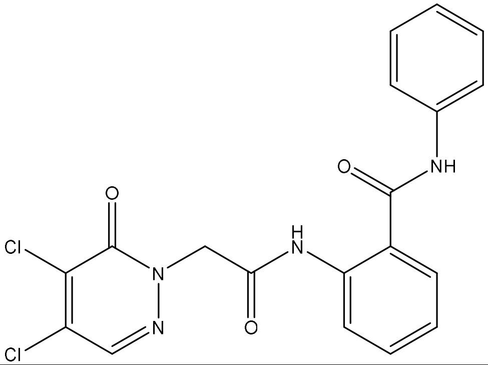	54.1	19.5	-11.2	17.3	5.77	0.8

#76	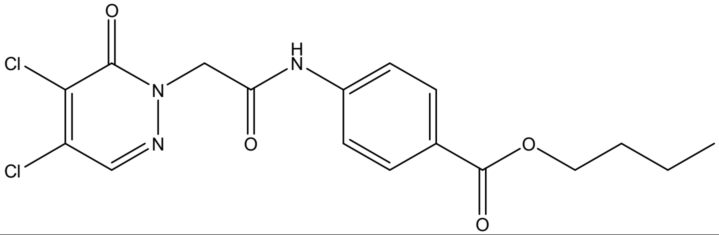	46.6	-4.4	-8.3	ND	ND	ND
#77	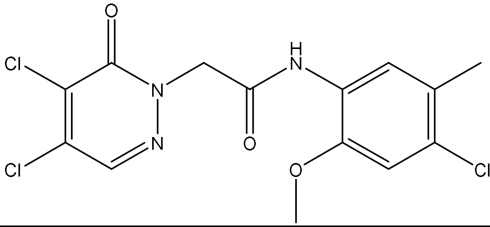	92.5	54.6	14.2	1.0	4.33	131.3
#78	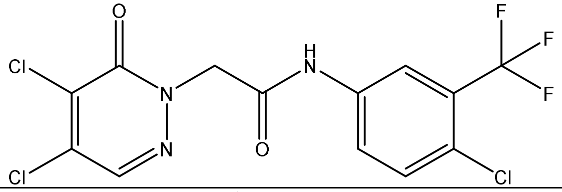	87.6	41.6	10.5	95.2	6.62	330.4
#79	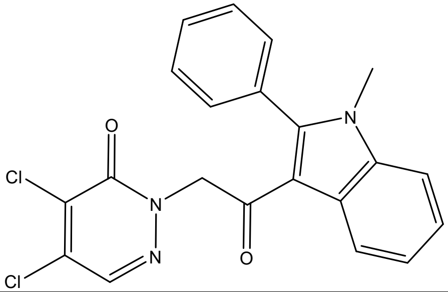	-52.3	-45.7	-52.1	ND	ND	ND

#80	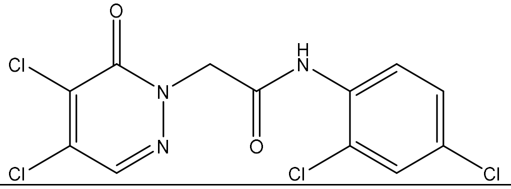	88.8	30.7	14.6	NA	NA	4.2
#81	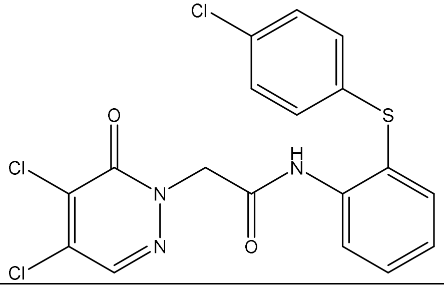	67.1	94.8	81.4	NA	NA	5.3
#82	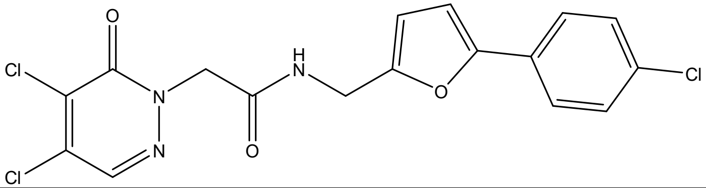	43.7	15.2	7.1	NA	NA	10.8
#83	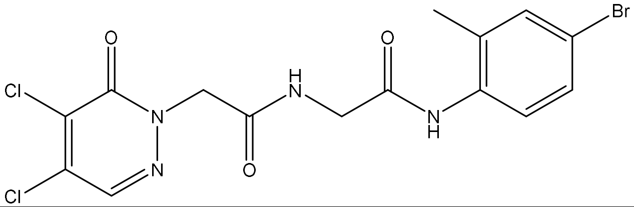	10.7	12.1	-0.8	NA	NA	-8.5
#84	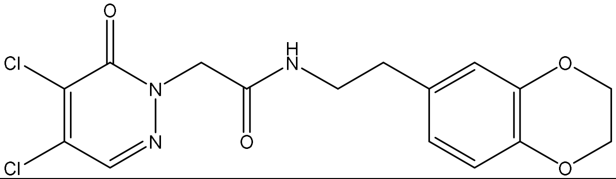	45.8	17.5	3.1	NA	NA	4.0

#85	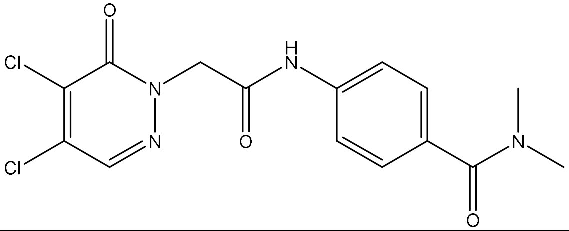	51.7	7.3	-9.7	NA	NA	-15.6
#86	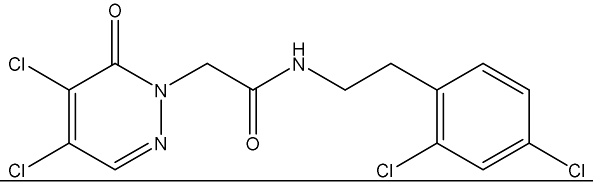	47.7	31.6	-1.9	NA	NA	-12.1
#87	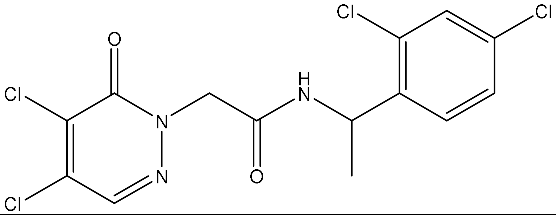	28.5	14.3	19.4	NA	NA	-2.1
#88	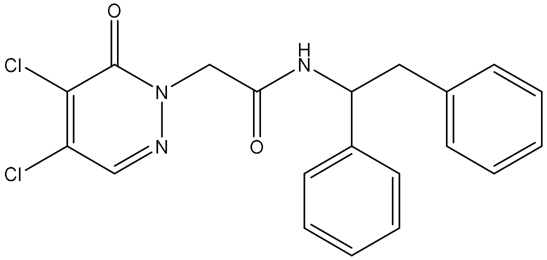	57.7	55.1	8.8	7.3	5.3	53.7

#89*	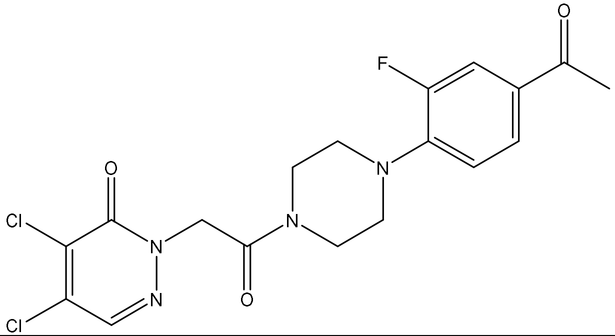	ND	ND	ND	ND	ND	ND
#90	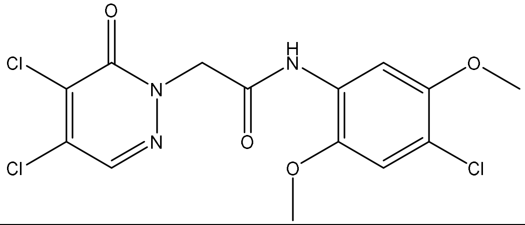	-14.3	2.6	10.5	ND	ND	ND
#91	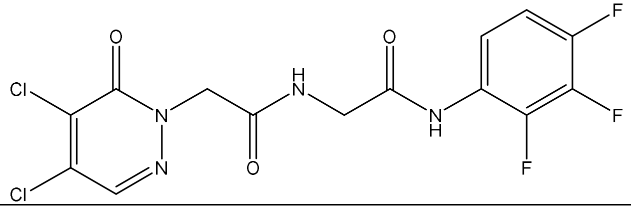	35.9	-14.3	11.6	19.0	4.34	6.3
#92	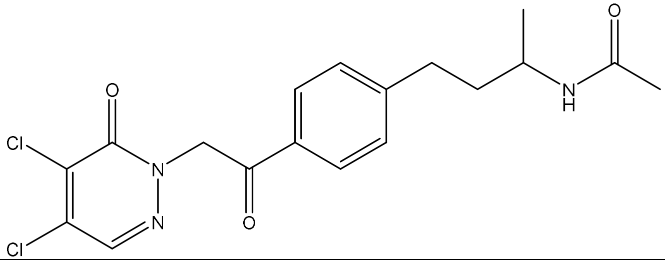	75.1	51.1	50.4	16.2	5.37	59.0

#93	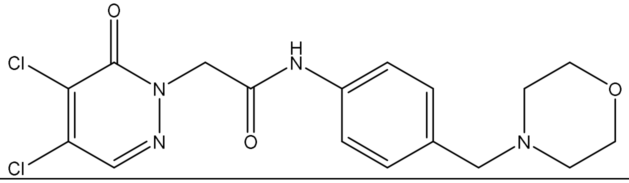	75.6	55.1	83.9	16.3	5.0	61.1
#94*	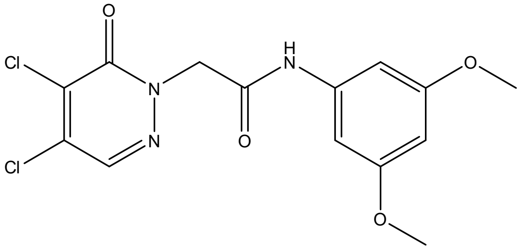	ND	ND	ND	ND	ND	ND
#95	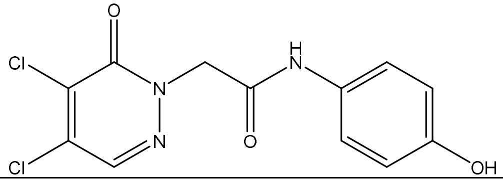	36.4	12.3	-13.3	14.4	4.36	2.4
#96	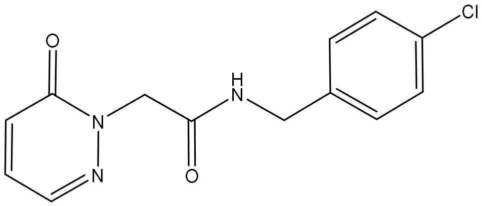	25.3	0.3	-10.0	ND	ND	ND
#97	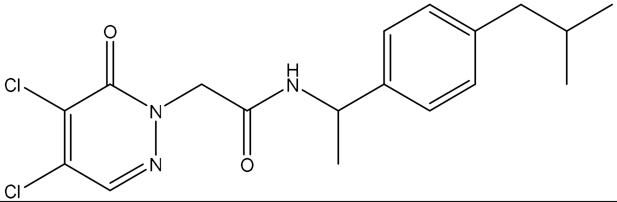	20.2	5.5	-10.6	ND	ND	ND

#98	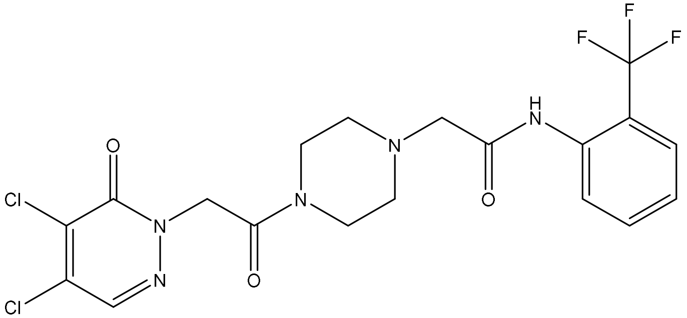	-20.0	4.1	15.5	ND	ND	ND
#99	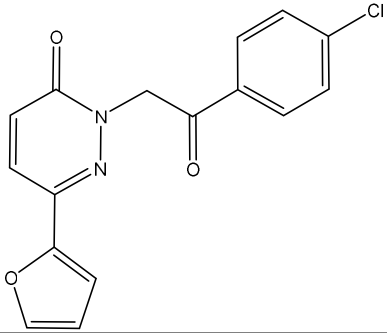	7.0	-1.8	-1.1	ND	ND	ND
#100	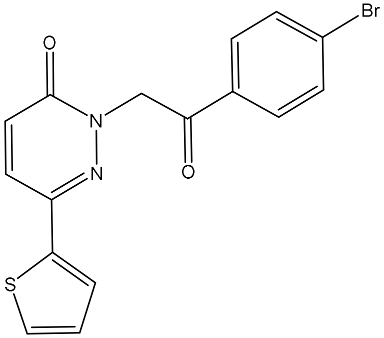	-9.1	6.5	0.6	ND	ND	ND

#101	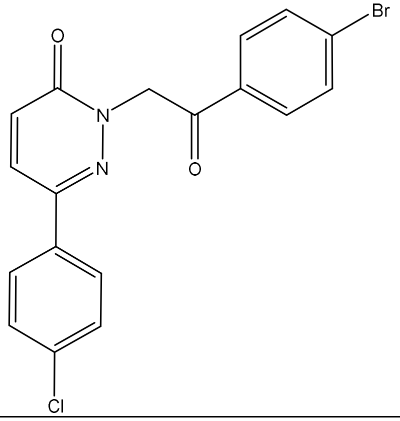	-4.3	-20.0	-1.4	ND	ND	ND
#102*	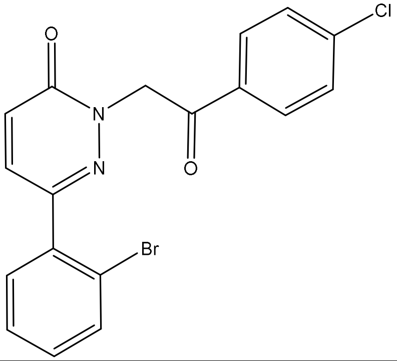	ND	ND	ND	ND	ND	ND

#103	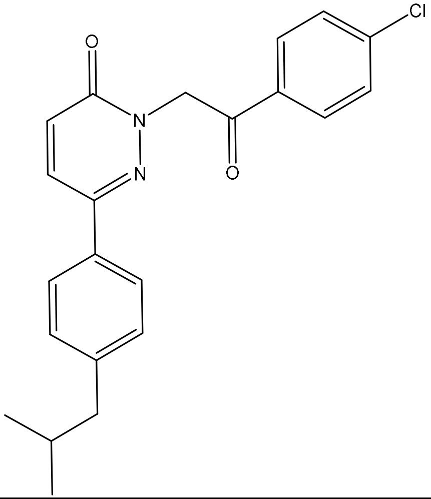	6.0	4.2	1.1	ND	ND	ND
#104*	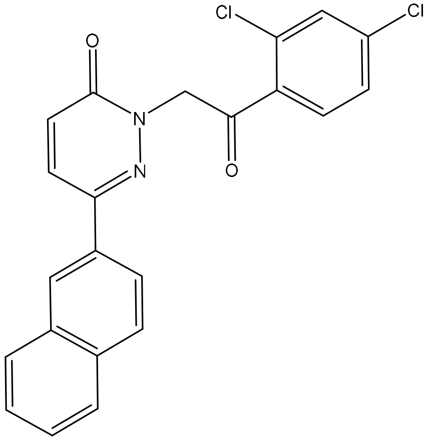	ND	ND	ND	ND	ND	ND

Activity, % above control: BNP-mediated (3/40/300nM rat BNP) cGMP production in presence of 10 µM compound
Efficacy, %: Efficacy of compound in presence of 3nM rat BNP. Calculated by construction of concentration-response curves of compound.
BNP efficacy, % above control: Efficacy of BNP in presence of 10 µM compound. Calculated by construction of concentration-response curves of BNP.
-logEC_{50}: -logEC_{50} of compound in presence of 3 nM rat BNP. Calculated by construction of concentration-response curves of compound.

NA: No activity. **ND:** Not determined.
*Compound was not tested due to solubility problems

Supporting Table 11. a. Overview of primers used in constructing chimeric GC-A/B. The pcDNA3.1(+) vector was linearized by restriction enzymes HindIII and XbaI and isolated from hGC-A or hGC-B pcDNA3.1(+) plasmids. Two or three DNA fragments were fused together with the linearized vector using the In-Fusion HD Enzyme premix that recognize a 15-20 bp overlap in the ends of each fragment. This overlap were added to the PCR primers.

Chimeric receptor	Primers for GC-A fragment(s)	Primers for GC-B fragment(s)
GC-A$^{1-462}$/B$^{447-1047}$	Forward: GCTAGCGTTTAAACTTAAGCTTACCATGCCCGGCCCCCGTAG<br>Reverse: GGTTTTGTCACAGCTAGGATCCTCATTGTCGAACCCAC	Forward: GGTTCGACAATGAGGATCCTAGCTGTGACAAAACCCCTCTGTC<br>Reverse: CAGCGGGTTTAAACGGGCCCTCTAGACTCGAGTTACAGCAGGCCG
GC-B$^{1-446}$/A$^{463-1061}$	Forward: CATTCGATCTCGACGACCCCGCTTGCAACCAGGATCATCT<br>Reverse: GGTTTAAACGGGCCCTCTAGACTATCCGCGCGTGCTACTG	Forward: TAGCGTTTAAACTTAAGCTTACCATGGCCCTGCCGTCTTT<br>Reverse: AGATGATCCTGGTTGCAAGCGGGGTCGTCGAGATCGAATG
GC-A$^{1-494}$/B$^{479-1047}$	Forward: GCTAGCGTTTAAACTTAAGCTTACCATGCCCGGCCCCCGTAG<br>Reverse: TTTTCGAGCATAAGTTTTCGGTAAATAAAGAAACTGACGA	Forward: TCGTCAGTTTCTTTATTTACCGAAAACTTATGCTCGAAAA<br>Reverse: CAGCGGGTTTAAACGGGCCCTCTAGACTCGAGTTACAGCAGGCCG
GC-B$^{1-478}$/A$^{495-1061}$	Forward: TCAGCTCCTTCCTGATTTTTCGCAAGATGCAGCTGGAGAA<br>Reverse: GGTTTAAACGGGCCCTCTAGACTATCCGCGCGTGCTACTG	Forward: TAGCGTTTAAACTTAAGCTTACCATGGCCCTGCCGTCTTT<br>Reverse: TTCTCCAGCTGCATCTTGCGAAAAATCAGGAAGGAGCTGA
GC-A$^{1-527}$/B$^{513-1047}$	Forward: GCTAGCGTTTAAACTTAAGCTTACCATGCCCGGCCCCCGTAG<br>Reverse: AGGGACAGAGTGAGCCGAGAGGCAGATCTCAGATGCCGCT	Forward: AGCGGCATCTGAGATCTGCCTCTCGGCTCACTCTGTCCCT<br>Reverse: CAGCGGGTTTAAACGGGCCCTCTAGACTCGAGTTACAGCAGGCCG
GC-B$^{1-512}$/A$^{528-1061}$	Forward: GCTATCATAAGGGAGCCGGAGGGAGCCGCCTGACTCTCTC<br>Reverse: GGTTTAAACGGGCCCTCTAGACTATCCGCGCGTGCTACTG	Forward: TAGCGTTTAAACTTAAGCTTACCATGGCCCTGCCGTCTTT<br>Reverse: GAGAGAGTCAGGCGGCTCCCTCCGGCTCCCTTATGATAGC
GC-A$^{1-805}$/B$^{787-1047}$	Forward: GCTAGCGTTTAAACTTAAGCTTACCATGCCCGGCCCCCGTAG<br>Reverse: CCCTCCTTATTAAAACGGCGGTTGAACTTCCTCAGGGTCA	Forward: TGACCCTGAGGAAGTTCAACCGCCGTTTTAATAAGGAGGG<br>Reverse: CAGCGGGTTTAAACGGGCCCTCTAGACTCGAGTTACAGCAGGCCG
GC-B$^{1-786}$/A$^{806-1061}$	Forward: GGCAGATCAAGGGTTTTATTCGGGAGAATTCCTCTAATAT<br>Reverse: GGTTTAAACGGGCCCTCTAGACTATCCGCGCGTGCTACTG	Forward: TAGCGTTTAAACTTAAGCTTACCATGGCCCTGCCGTCTTT<br>Reverse: ATATTAGAGGAATTCTCCCGAATAAAACCCTTGATCTGCC
GC-A$^{1-875}$/B$^{861-1047}$	Forward: GCTAGCGTTTAAACTTAAGCTTACCATGCCCGGCCCCCGTAG<br>Reverse: ATATCGGAGAAGTAGATGGTGACTGAGTCAAAGGCTTCGG	Forward: CCGAAGCCTTTGACTCAGTCACCATCTACTTCTCCGATAT<br>Reverse: CAGCGGGTTTAAACGGGCCCTCTAGACTCGAGTTACAGCAGGCCG
GC-B$^{1-860}$/A$^{876-1061}$	Forward: CTGAGGCCTTTGACTCAGTGACTATCTATTTTTCCGACAT<br>Reverse: GGTTTAAACGGGCCCTCTAGACTATCCGCGCGTGCTACTG	Forward: TAGCGTTTAAACTTAAGCTTACCATGGCCCTGCCGTCTTT<br>Reverse: ATGTCGGAAAAATAGATAGTCACTGAGTCAAAGGCCTCAG
GC-A$^{1-462}$/B$^{447-458}$/A$^{474-1061}$	1. Forward: GCTAGCGTTTAAACTTAAGCTTACCATGCCCGGCCCCCGTAG<br>Reverse: AGAGGGGTTTTGTCACAGCTAGGATCCTCATTGTCGAACC<br>2. Forward: CTCTGTCCACACTCGCCATCGTGCTTGCACTGGTTGGCTC<br>Reverse: GGTTTAAACGGGCCCTCTAGACTATCCGCGCGTGCTACTG	Forward: GGTTCGACAATGAGGATCCTAGCTGTGACAAAACCCCTCT<br>Reverse: GAGCCAACCAGTGCAAGCACGATGGCGAGTGTGGACAGAG
GC-B$^{1-446}$/A$^{463-473}$/B$^{459-1047}$	Forward: CATTCGATCTCGACGACCCCGCTTGCAACCAGGATCATCT<br>Reverse: ATGCCGGTGCCGAGTGCGACTTCCAAGGTACTAAGATGAT	1. Forward: TAGCGTTTAAACTTAAGCTTACCATGGCCCTGCCGTCTTT<br>Reverse: AGATGATCCTGGTTGCAAGCGGGGTCGTCGAGATCGAATG<br>2. Forward: ATCATCTTAGTACCTTGGAAGTCGCACTCGGCACCGGCAT<br>Reverse: CAGCGGGTTTAAACGGGCCCTCTAGACTCGAGTTACAGCAGGCCG
GC-A$^{1-473}$/B$^{459-478}$/A$^{495-1061}$	1. Forward: GCTAGCGTTTAAACTTAAGCTTACCATGCCCGGCCCCCGTAG	Forward: ATCATCTTAGTACCTTGGAAGTCGCACTCGGCACCGGCAT<br>Reverse: TTCTCCAGCTGCATCTTGCGAAAAATCAGGAAGGAGCTGA

	Reverse: ATGCCGGTGCCGAGTGCGACTTCCAAGGTACTAAGATGAT<br>2. Forward: TCAGCTCCTTCCTGATTTTTCGCAAGATGCAGCTGGAGAA<br>Reverse: GGTTTAAACGGGCCCTCTAGACTATCCGCGCGTGCTACTG	
GC-B^{1-458}/$A^{474-494}$/$B^{479-1047}$	Forward: CTCTGTCCACACTCGCCATCGTGCTTGCACTGGTTGGCTC<br>Reverse: TTTTCGAGCATAAGTTTTCGGTAAATAAAGAAACTGACGA	1. Forward: TAGCGTTTAAACTTAAGCTTACCATGGCCCTGCCGTCTTT<br>Reverse: GAGCCAACCAGTGCAAGCACGATGGCGAGTGTGGACAGAG<br>2. Forward: TCGTCAGTTTCTTTATTTACCGAAAACTTATGCTCGAAAA<br>Reverse: CAGCGGGTTTAAACGGGCCCTCTAGACTCGAGTTACAGCAGGCCG
GC-A^{1-527}/$B^{513-786}$/$A^{806-1061}$	1. Forward: GCTAGCGTTTAAACTTAAGCTTACCATGCCCGGCCCCCGTAG<br>Reverse: AGGGACAGAGTGAGCCGAGA GGCAGATCTCAGATGCCGCT<br>2. Forward: GGCAGATCAAGGGTTTTATTCGGGAGAATTCCTCTAATAT<br>Reverse: GGTTTAAACGGGCCCTCTAGACTATCCGCGCGTGCTACTG	Forward: AGCGGCATCTGAGATCTGCCTCTCGGCTCACTCTGTCCCT<br>Reverse: ATATTAGAGGAATTCTCCCGAATAAAACCCTTGATCTGCC
GC-B^{1-512}/$A^{528-805}$/$B^{787-1047}$	Forward: GCTATCATAAGGGAGCCGGAGGGAGCCGCCTGACTCTCTC<br>Reverse: CCCTCCTTATTAAAACGGCGGTTGAACTTCCTCAGGGTCA	1. Forward: TAGCGTTTAAACTTAAGCTTACCATGGCCCTGCCGTCTTT<br>Reverse: GAGAGAGTCAGGCGGCTCCCTCCGGCTCCCTTATGATAGC<br>2. Forward: TGACCCTGAGGAAGTTCAACCGCCGTTTTAATAAGGAGGG<br>Reverse: CAGCGGGTTTAAACGGGCCCTCTAGACTCGAGTTACAGCAGGCCG
GC-A^{1-805}/$B^{787-860}$/$A^{876-1061}$	1. Forward: GCTAGCGTTTAAACTTAAGCTTACCATGCCCGGCCCCCGTAG<br>Reverse: CCCTCCTTATTAAAACGGCGGTTGAACTTCCTCAGGGTCA<br>2. Forward: CTGAGGCCTTTGACTCAGTGACTATCTATTTTTCCGACAT<br>Reverse: GGTTTAAACGGGCCCTCTAGACTATCCGCGCGTGCTACTG	Forward: TGACCCTGAGGAAGTTCAACCGCCGTTTTAATAAGGAGGG<br>Reverse: ATGTCGGAAAAATAGATAGTCACTGAGTCAAAGGCCTCAG
GC-B^{1-786}/$A^{806-875}$/$B^{861-1047}$	Forward: GGCAGATCAAGGGTTTTATTCGGGAGAATTCCTCTAATAT<br>Reverse: ATATCGGAGAAGTAGATGGTGACTGAGTCAAAGGCTTCGG	1. Forward: TAGCGTTTAAACTTAAGCTTACCATGGCCCTGCCGTCTTT<br>Reverse: ATATTAGAGGAATTCTCCCGAATAAAACCCTTGATCTGCC<br>2. Forward: CCGAAGCCTTTGACTCAGTCACCATCTACTTCTCCGATAT<br>Reverse: CAGCGGGTTTAAACGGGCCCTCTAGACTCGAGTTACAGCAGGCCG
GC-A^{1-527}/$B^{513-860}$/$A^{876-1061}$	1. Forward: GCTAGCGTTTAAACTTAAGCTTACCATGCCCGGCCCCCGTAG<br>Reverse: AGGGACAGAGTGAGCCGAGAGGCAGATCTCAGATGCCGCT<br>2. Forward: CTGAGGCCTTTGACTCAGTGACTATCTATTTTTCCGACAT<br>Reverse: GGTTTAAACGGGCCCTCTAGACTATCCGCGCGTGCTACTG	Forward: AGCGGCATCTGAGATCTGCCTCTCGGCTCACTCTGTCCCT<br>Reverse: ATGTCGGAAAAATAGATAGTCACTGAGTCAAAGGCCTCAG
GC-B^{1-512}/$A^{528-875}$/$B^{861-1047}$	Forward: GCTATCATAAGGGAGCCGGAGGGAGCCGCCTGACTCTCTC<br>Reverse: ATATCGGAGAAGTAGATGGTGACTGAGTCAAAGGCTTCGG	1. Forward: TAGCGTTTAAACTTAAGCTTACCATGGCCCTGCCGTCTTT<br>Reverse: GAGAGAGTCAGGCGGCTCCCTCCGGCTCCCTTATGATAGC<br>2. Forward: CCGAAGCCTTTGACTCAGTCACCATCTACTTCTCCGATAT<br>Reverse: CAGCGGGTTTAAACGGGCCCTCTAGACTCGAGTTACAGCAGGCCG
GC-A^{1-512}/$B^{497-1047}$	Forward: GCTAGCGTTTAAACTTAAGCTTACCATGCCCGGCCCCCGTAG<br>Reverse: TTTCCGAACTGCAATTCCTCCCACCTAACACGCCACAGCT	Forward: AGCTGTGGCGTGTTAGGTGGGAGGAATTGCAGTTCGGAAA<br>Reverse: CAGCGGGTTTAAACGGGCCCTCTAGACTCGAGTTACAGCAGGCCG
GC-B^{1-496}/$A^{513-1061}$	Forward: TGCTTTGGCGAATCAGGTGGGAGGATGTGGAACCATCAAG<br>Reverse: GGTTTAAACGGGCCCTCTAGACTATCCGCGCGTGCTACTG	Forward: TAGCGTTTAAACTTAAGCTTACCATGGCCCTGCCGTCTTT<br>Reverse: CTTGATGGTTCCACATCCTCCCACCTGATTCGCCAAAGCA
GC-A^{1-528}/$B^{513-1047}$	Forward: GCTAGCGTTTAAACTTAAGCTTACCATGCCCGGCCCCCGTAG<br>Reverse: AGGGACAGAGTGAGCCGAGACCCGGCAGATCTCAGATGCC	Forward: GGCATCTGAGATCTGCCGGGTCTCGGCTCACTCTGTCCCT<br>Reverse: CAGCGGGTTTAAACGGGCCCTCTAGACTCGAGTTACAGCAGGCCG
GC-B^{1-512}/$A^{529-1061}$	Forward: GCTATCATAAGGGAGCCGGAAGCCGCCTGACTCTCTCCGG<br>Reverse: GGTTTAAACGGGCCCTCTAGACTATCCGCGCGTGCTACTG	Forward: TAGCGTTTAAACTTAAGCTTACCATGGCCCTGCCGTCTTT<br>Reverse: CCGGAGAGAGTCAGGCGGCTTCCGGCTCCCTTATGATAGC
GC-A^{1-573}/$B^{558-1047}$	Forward: GCTAGCGTTTAAACTTAAGCTTACCATGCCCGGCCCCCGTAG<br>Reverse: ACCTGACGAGTAAGCTCGATCCGCTTGCGGTTCACCCGCT	Forward: AGCGGGTGAACCGCAAGCGGATCGAGCTTACTCGTCAGGT<br>Reverse: CAGCGGGTTTAAACGGGCCCTCTAGACTCGAGTTACAGCAGGCCG
GC-B^{1-557}/$A^{574-1061}$	Forward: AGCACGTGAATAAGAAGCGGATTGAGCTGACGCGCAAAGT<br>Reverse: GGTTTAAACGGGCCCTCTAGACTATCCGCGCGTGCTACTG	Forward: TAGCGTTTAAACTTAAGCTTACCATGGCCCTGCCGTCTTT<br>Reverse: ACTTTGCGCGTCAGCTCAATCCGCTTCTTATTCACGTGCT

GC-A$^{1-620}$/B$^{605-1047}$	Forward: GCTAGCGTTTAAACTTAAGCTTACCATGCCCGGCCCCCGTAG<br>Reverse: TCGTTTTCCAGAATATCCTGGAGAGAGCCCCGTGGGCAAT	Forward: ATTGCCCACGGGGCTCTCTCCAGGATATTCTGGAAAACGA<br>Reverse: CAGCGGGTTTAAACGGGCCCTCTAGACTCGAGTTACAGCAGGCCG
GC-B$^{1-604}$/A$^{621-1061}$	Forward: ATTGTCCGCGGGGAAGTTTGCAGGACATTCTCGAGAACGA<br>Reverse: GGTTTAAACGGGCCCTCTAGACTATCCGCGCGTGCTACTG	Forward: TAGCGTTTAAACTTAAGCTTACCATGGCCCTGCCGTCTTT<br>Reverse: TCGTTCTCGAGAATGTCCTGCAAACTTCCCCGCGGACAAT
GC-A$^{1-663}$/B$^{648-1047}$	Forward: GCTAGCGTTTAAACTTAAGCTTACCATGCCCGGCCCCCGTAG<br>Reverse: CTGTCAACCACACAGTTAGATGACTTAAGGTTTCCGTGGG	Forward: CCCACGGAAACCTTAAGTCATCTAACTGTGTGGTTGACAG<br>Reverse: CAGCGGGTTTAAACGGGCCCTCTAGACTCGAGTTACAGCAGGCCG
GC-B$^{1-647}$/A$^{664-1061}$	Forward: CACACGGATCACTTAAAAGCTCAAACTGTGTGGTGGACGG<br>Reverse: GGTTTAAACGGGCCCTCTAGACTATCCGCGCGTGCTACTG	Forward: TAGCGTTTAAACTTAAGCTTACCATGGCCCTGCCGTCTTT<br>Reverse: CCGTCCACCACACAGTTTGAGCTTTTAAGTGATCCGTGTG
GC-A$^{1-700}$/B$^{686-1047}$	Forward: GCTAGCGTTTAAACTTAAGCTTACCATGCCCGGCCCCCGTAG<br>Reverse: AGCAACTCCGGGGCGGTCCACAGCTTCTTTGCGTAGACGG	Forward: CCGTCTACGCAAAGAAGCTGTGGACCGCCCCGGAGTTGCT<br>Reverse: CAGCGGGTTTAAACGGGCCCTCTAGACTCGAGTTACAGCAGGCCG
GC-B$^{1-685}$/A$^{701-1061}$	Forward: CCCTGTACGCCAAGAAGCTGTGGACCGCTCCCGAGCTGCT<br>Reverse: GGTTTAAACGGGCCCTCTAGACTATCCGCGCGTGCTACTG	Forward: TAGCGTTTAAACTTAAGCTTACCATGGCCCTGCCGTCTTT<br>Reverse: AGCAGCTCGGGAGCGGTCCACAGCTTCTTGGCGTACAGGG
GC-A$^{1-729}$/B$^{715-1047}$	Forward: GCTAGCGTTTAAACTTAAGCTTACCATGCCCGGCCCCCGTAG<br>Reverse: GAGCGCAGAGCAATTTCCTGCAGTATGATCCCGAAGGAGT	Forward: ACTCCTTCGGGATCATACTGCAGGAAATTGCTCTGCGCTC<br>Reverse: CAGCGGGTTTAAACGGGCCCTCTAGACTCGAGTTACAGCAGGCCG
GC-B$^{1-714}$/A$^{730-1061}$	Forward: ACTCCTTTGGAATTATCCTGCAGGAAATTGCTCTTCGGAG<br>Reverse: GGTTTAAACGGGCCCTCTAGACTATCCGCGCGTGCTACTG	Forward: TAGCGTTTAAACTTAAGCTTACCATGGCCCTGCCGTCTTT<br>Reverse: CTCCGAAGAGCAATTTCCTGCAGGATAATTCCAAAGGAGT
GC-A$^{1-620}$/B$^{605-714}$/A$^{730-1061}$	1. Forward: GCTAGCGTTTAAACTTAAGCTTACCATGCCCGGCCCCCGTAG<br>Reverse: TCGTTTTCCAGAATATCCTGGAGAGAGCCCCGTGGGCAAT<br>2. Forward: ACTCCTTTGGAATTATCCTGCAGGAAATTGCTCTTCGGAG<br>Reverse: GGTTTAAACGGGCCCTCTAGACTATCCGCGCGTGCTACTG	Forward: ATTGCCCACGGGGCTCTCTCCAGGATATTCTGGAAAACGA<br>Reverse: CTCCGAAGAGCAATTTCCTGCAGGATAATTCCAAAGGAGT
GC-B$^{1-604}$/A$^{621-729}$/B$^{715-1047}$	Forward: ATTGTCCGCGGGGAAGTTTGCAGGACATTCTCGAGAACGA<br>Reverse: GAGCGCAGAGCAATTTCCTGCAGTATGATCCCGAAGGAGT	1. Forward: TAGCGTTTAAACTTAAGCTTACCATGGCCCTGCCGTCTTT<br>Reverse: TCGTTCTCGAGAATGTCCTGCAAACTTCCCCGCGGACAAT<br>2. Forward: ACTCCTTCGGGATCATACTGCAGGAAATTGCTCTGCGCTC<br>Reverse: CAGCGGGTTTAAACGGGCCCTCTAGACTCGAGTTACAGCAGGCCG
GC-A$^{1-620}$/B$^{605-647}$/A$^{664-1061}$	1. Forward: GCTAGCGTTTAAACTTAAGCTTACCATGCCCGGCCCCCGTAG<br>Reverse: TCGTTTTCCAGAATATCCTGGAGAGAGCCCCGTGGGCAAT<br>2. Forward: CACACGGATCACTTAAAAGCTCAAACTGTGTGGTGGACGG<br>Reverse: GGTTTAAACGGGCCCTCTAGACTATCCGCGCGTGCTACTG	Forward: ATTGCCCACGGGGCTCTCTCCAGGATATTCTGGAAAACGA<br>Reverse: CCGTCCACCACACAGTTTGAGCTTTTAAGTGATCCGTGTG
GC-B$^{1-604}$/A$^{621-663}$/B$^{648-1047}$	Forward: ATTGTCCGCGGGGAAGTTTGCAGGACATTCTCGAGAACGA<br>Reverse: CTGTCAACCACACAGTTAGATGACTTAAGGTTTCCGTGGG	1. Forward: TAGCGTTTAAACTTAAGCTTACCATGGCCCTGCCGTCTTT<br>Reverse: TCGTTCTCGAGAATGTCCTGCAAACTTCCCCGCGGACAAT<br>2. Forward: CCCACGGAAACCTTAAGTCATCTAACTGTGTGGTTGACAG<br>Reverse: CAGCGGGTTTAAACGGGCCCTCTAGACTCGAGTTACAGCAGGCCG
GC-A$^{1-663}$/B$^{648-686}$/A$^{701-1061}$	1. Forward: GCTAGCGTTTAAACTTAAGCTTACCATGCCCGGCCCCCGTAG<br>Reverse: CTGTCAACCACACAGTTAGATGACTTAAGGTTTCCGTGGG<br>2. Forward: CCCTGTACGCCAAGAAGCTGTGGACCGCTCCCGAGCTGCT<br>Reverse: GGTTTAAACGGGCCCTCTAGACTATCCGCGCGTGCTACTG	Forward: CCCACGGAAACCTTAAGTCATCTAACTGTGTGGTTGACAG<br>Reverse: AGCAGCTCGGGAGCGGTCCACAGCTTCTTGGCGTACAGGG
GC-B$^{1-647}$/A$^{664-700}$/B$^{686-1047}$	Forward: CACACGGATCACTTAAAAGCTCAAACTGTGTGGTGGACGG<br>Reverse: AGCAACTCCGGGGCGGTCCACAGCTTCTTTGCGTAGACGG	1. Forward: TAGCGTTTAAACTTAAGCTTACCATGGCCCTGCCGTCTTT<br>Reverse: CCGTCCACCACACAGTTTGAGCTTTTAAGTGATCCGTGTG<br>2. Forward: CCGTCTACGCAAAGAAGCTGTGGACCGCCCCGGAGTTGCT<br>Reverse: CAGCGGGTTTAAACGGGCCCTCTAGACTCGAGTTACAGCAGGCCG
GC-A$^{1-700}$/B$^{686-715}$/A$^{731-1061}$	1. Forward: GCTAGCGTTTAAACTTAAGCTTACCATGCCCGGCCCCCGTAG<br>Reverse: AGCAACTCCGGGGCGGTCCACAGCTTCTTTGCGTAGACGG<br>2. Forward: ACTCCTTTGGAATTATCCTGCAGGAAATTGCTCTTCGGAG<br>Reverse: GGTTTAAACGGGCCCTCTAGACTATCCGCGCGTGCTACTG	Forward: CCGTCTACGCAAAGAAGCTGTGGACCGCCCCGGAGTTGCT<br>Reverse: CTCCGAAGAGCAATTTCCTGCAGGATAATTCCAAAGGAGT

GC-B$^{1-685}$/A$^{701-730}$/B$^{716-1047}$	Forward: CCCTGTACGCCAAGAAGCTGTGGACCGCTCCCGAGCTGCT<br>Reverse: GAGCGCAGAGCAATTTCCTGCAGTATGATCCCGAAGGAGT	1. Forward: TAGCGTTTAAACTTAAGCTTACCATGGCCCTGCCGTCTTT<br>Reverse: AGCAGCTCGGGAGCGGTCCACAGCTTCTTGGCGTACAGGG<br>2. Forward: ACTCCTTCGGGATCATACTGCAGGAAATTGCTCTGCGCTC<br>Reverse: CAGCGGGTTTAAACGGGCCCTCTAGACTCGAGTTACAGCAGGCCG

b. Overview of primers used in constructing single or dual mutations in GC-A and GC-B. Primers for mutagenesis were mainly design using the web based In-Fusion Cloning Primer Design Tool from Takara Bio (available from https://www.takarabio.com/learning-centers/cloning/primer-design-and-other-tools). The primers have a 15 bp overlap in the 5' end and the mutation incorporated. GC-A or GC-B was used as templates unless otherwise is specified.

**Construct**	**Primers**
GC-A^{E627D}	Forward: CGAGAACGACTCCATTACACTGGACTGGATGTTCC<br>Reverse: ATGGAGTCGTTCTCGAGAATGTCCTGGAGAG
GC-A^{E627D_T630N}	Using GC-A^{E627D} as template:<br>Forward: CTCCATTAACCTGGACTGGATGTTCCGC<br>Reverse: TCCAGGTTAATGGAGTCGTTCTCGAGAATGTC
GC-A^{T640I}	Forward: TAGTCTGATTAATGACATCGTCAAGGGGATGC<br>Reverse: TCATTAATCAGACTATAGCGGAACATCCAGTCC
GC-A^{I643L}	Forward: TAATGACCTTGTCAAGGGGATGCTCTTCC<br>Reverse: TTGACAAGGTCATTAGTCAGACTATAGCGGAAC
GC-A^{T640I_I643L}	Forward: TAGTCTGATTAATGACCTTGTCAAGGGGATGCTCTTCC<br>Reverse: TTGACAAGGTCATTAATCAGACTATAGCGGAACATCCAGT
GC-A^{L648A}	Forward: GGGGATGGCTTTCCTTCACAACGGTGCC<br>Reverse: AGGAAAGCCATCCCCTTGACGATGTCATTAGTC
GC-A^{G653S}	Forward: TCACAACTCCGCCATCTGCTCCCACGGA<br>Reverse: ATGGCGGAGTTGTGAAGGAAGAGCATCCCCT
GC-A^{G653S_A654I}	Using GC-A^{G653S} as template:<br>Forward: CAACTCCATTATCTGCTCCCACGGAAACC<br>Reverse: CAGATAATGGAGTTGTGAAGGAAGAGCATCC
GC-A^{C656S_N660S}	First constructed GC-A^{C656S} using<br>Forward: TGCCATCTCATCCCACGGAAACCTTAAGTC<br>Reverse: TGGGATGAGATGGCACCGTTGTGAAGG<br>Then constructed GC-A^{C656S_N660S} using GC-A^{C656S} as the template and primers:<br>Forward: CCACGGATCACTTAAGTCATCAAACTGTGTGGTGG<br>Reverse: TTAAGTGATCCGTGGGATGAGATGGC
GC-B^{D611E}	Forward: GGAAAACGAGAGCATCAACCTTGATTGGATGTTC<br>Reverse: ATGCTCTCGTTTTCCAGAATATCCTGCAAAC
GC-B^{D611E_N614T}	Using GC-B^{D611E} as template:<br>Forward: GAGCATCACACTTGATTGGATGTTCCGCTACTCC<br>Reverse: TCAAGTGTGATGCTCTCGTTTTCCAGAATATCC

GC-B^{I624T}	Forward: CTCCCTGACTAACGACCTTGTGAAAGGTATGGCT<br>Reverse: TCGTTAGTCAGGGAGTAGCGGAACATCCAA
GC-B^{L627I}	Forward: TAACGACATCGTGAAAGGTATGGCTTTCCTGC<br>Reverse: TTCACGATGTCGTTAATCAGGGAGTAGCGG
GC-B^{I624T_L627I}	Forward: CTCCCTGACTAACGACATCGTGAAAGGTATGGCTTTCCTG<br>Reverse: TTCACGATGTCGTTAGTCAGGGAGTAGCGGAACATCCAA
GC-B^{A632L}	Forward: AGGTATGCTGTTCCTGCACAATTCCATTATCTCA<br>Reverse: AGGAACAGCATACCTTTCACAAGGTCGTT
GC-B^{S637G}	Forward: GCACAATGGTATTATCTCATCACACGGATCACT<br>Reverse: ATAATACCATTGTGCAGGAAAGCCATACCT
GC-B^{S637G_I638A}	Using GC-B^{S637G} as template:<br>Forward: CAATGGTGCCATCTCATCACACGGATCACTT<br>Reverse: GAGATGGCACCATTGTGCAGGAAAGCCA
GC-B^{S640C_S644N}	First constructed GC-B^{S640C} using<br>Forward: CATTATCTGCTCACACGGATCACTTAAAAGC<br>Reverse: TGTGAGCAGATAATGGAATTGTGCAGGAAAGC<br>Then constructed GC-B^{S640C_S644N} using GC-B^{S640C} as the template and primers:<br>Forward: ACACGGAAACCTTAAAAGCTCTAACTGTGTGGTTG<br>Reverse: TTAAGGTTTCCGTGTGAGCAGATAATGG
**GC-AT640 and GC-BI624 focused constructs**	
GC-A^{T640A}	Forward: TAGTCTGGCTAATGACATCGTCAAGGGGATGC<br>Reverse: TCATTAGCCAGACTATAGCGGAACATCCAGTCC
GC-A^{T640D}	Forward: TAGTCTGGATAATGACATCGTCAAGGGGATGC<br>Reverse: TCATTATCCAGACTATAGCGGAACATCCAGTCC
GC-A^{T640E}	Forward: TAGTCTGGAGAATGACATCGTCAAGGGGATGC<br>Reverse: TCATTCTCCAGACTATAGCGGAACATCCAGTCC
GC-A^{T640L}	Forward: TAGTCTGCTTAATGACATCGTCAAGGGGATGC<br>Reverse: TCATTAAGCAGACTATAGCGGAACATCCAGTCC
GC-A^{T640S}	Forward: TAGTCTGAGTAATGACATCGTCAAGGGGATGC<br>Reverse: TCATTACTCAGACTATAGCGGAACATCCAGTCC
GC-A^{T640Y}	Forward: TAGTCTGTACAATGACATCGTCAAGGGGATGC<br>Reverse: TCATTGTACAGACTATAGCGGAACATCCAGTCC
GC-A^{T640V}	Forward: TAGTCTGGTTAATGACATCGTCAAGGGGATGC<br>Reverse: TCATTAACCAGACTATAGCGGAACATCCAGTCC
GC-B^{I624A}	Forward: CTCCCTGGCTAACGACCTTGTGAAAGGTATGGCT<br>Reverse: TCGTTAGCCAGGGAGTAGCGGAACATCCAA
GC-B^{I624D}	Forward: CTCCCTGGATAACGACCTTGTGAAAGGTATGGCT<br>Reverse: TCGTTATCCAGGGAGTAGCGGAACATCCAA
GC-B^{I624L}	Forward: CTCCCTGCTTAACGACCTTGTGAAAGGTATGGCT<br>Reverse: TCGTTAAGCAGGGAGTAGCGGAACATCCAA
GC-B^{I624S}	Forward: CTCCCTGAGTAACGACCTTGTGAAAGGTATGGCT<br>Reverse: TCGTTACTCAGGGAGTAGCGGAACATCCAA
GC-B^{I624Y}	Forward: CTCCCTGTACAACGACCTTGTGAAAGGTATGGCT<br>Reverse: TCGTTGTACAGGGAGTAGCGGAACATCCAA

GC-B^{I624V}	Forward: CTCCCTGGTTAACGACCTTGTGAAAGGTATGGCT<br>Reverse: TCGTTAACCAGGGAGTAGCGGAACATCCAA
**GC^{7E}**	
GC-A^{S519E}	Forward: ACCATCAGAGCTGGAGCGGCATCTGAGATCTG<br>Reverse: TCCAGCTCTGATGGTTCCACATCCTCCCAC
GC-A^{S519E_S529E}	Using GC-A^{S519E} as template:<br>Forward: TGCCGGGGAACGCCTGACTCTCTCCGGAC<br>Reverse: AGGCGTTCCCCGGCAGATCTCAGATGC
GC-A^{S519E_S529E_T532E}	Using GC-A^{S519E_S529E} as template:<br>Forward: ACGCCTGGAACTCTCCGGACGAGGCTCCA<br>Reverse: GAGAGTTCCAGGCGTTCCCCGGCAGA
GC-A^{S519E_S529E_T532E_S534E}	Using GC-A^{S519E_S529E_T532E} as template:<br>Forward: GGAACTCGAAGGACGAGGCTCCAACTACGG<br>Reverse: CGTCCTTCGAGTTCCAGGCGTTCCCC
GC-A^{S519E_S529E_T532E_S534E_S538E}	Using GC-A^{S519E_S529E_T532E_S534E} as template:<br>Forward: ACGAGGCGAGAACTACGGTTCTTTGCTGACAACC<br>Reverse: TAGTTCTCGCCTCGTCCTTCGAGTTCC
GC-_A^{S519E_S529E_T532E_S534E_S538E_S542E}	Using GC-A^{S519E_S529E_T532E_S534E_S538E} as template:<br>Forward: CTACGGTGAGTTGCTGACAACCGAAGGCCA<br>Reverse: AGCAACTCACCGTAGTTCTCGCCTCGT
GC-A^{S519E_S529E_T532E_S534E_S538E_S542E_T545E}	Using GC-_A^{S519E_S529E_T532E_S534E_S538E_S542E} as template:<br>Forward: GTTGCTGGAAACCGAAGGCCAGTTCCAGG<br>Reverse: TCGGTTTCCAGCAACTCACCGTAGTTCTCG
